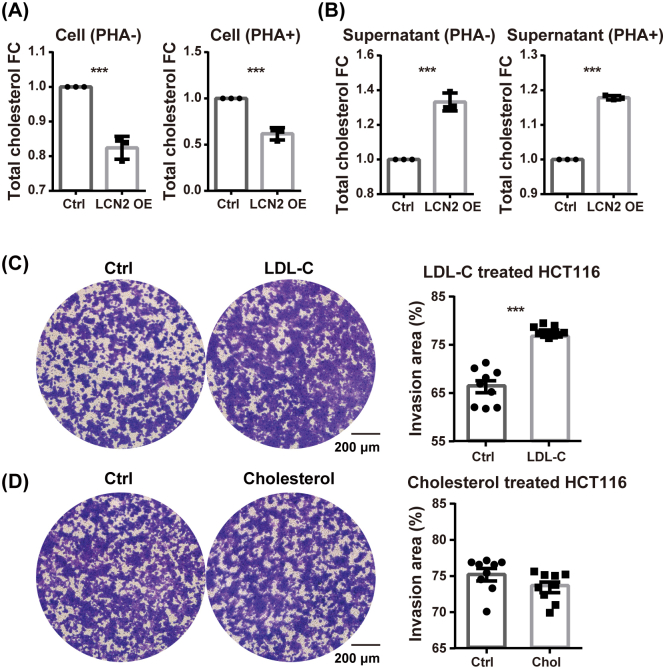# Quantitative Proteomics of Tissue-Infiltrating T Cells From CRC Patients Identified Lipocalin-2 Induces T-Cell Apoptosis and Promotes Tumor Cell Proliferation by Iron Efflux

**DOI:** 10.1016/j.mcpro.2023.100691

**Published:** 2023-12-10

**Authors:** Rui Che, Qingsong Wang, Minzhe Li, Jian Shen, Jianguo Ji

**Affiliations:** 1 State Key Laboratory of Protein and Plant Gene Research, School of Life Sciences, Peking University, Beijing, China; 2 General Surgery Department, Beijing Chao-Yang Hospital, Capital Medical University, Beijing, China

**Keywords:** lipocalin-2, colorectal cancer, tumor-infiltrating T cells, iron transport, antitumor immunity

## Abstract

T cells play the most pivotal roles in antitumor immunity; the T-cell proteome and the differentially expressed proteins in the tumor immune microenvironment have rarely been identified directly from the clinical samples, especially for tumors that lack effective immunotherapy targets, such as colorectal cancer (CRC). In this study, we analyzed the protein expression pattern of the infiltrating T cells isolated from CRC patients using quantitative proteomics. CD4^+^ and CD8^+^ T cells were isolated from clinical samples and labeled by tandem mass tag reagents, and the differentially expressed proteins were quantified by mass spectrometry. The T-cell proteome profiling revealed dysfunctions in these tumor-infiltrating T cells. Specifically, antitumor immunity was suppressed because of differentially expressed metal ion transporters and immunity regulators. For the first time, lipocalin-2 (LCN2) was shown to be significantly upregulated in CD4^+^ T cells. Quantitative proteomic analysis of LCN2-overexpressed Jurkat cells showed that LCN2 damaged T cells by changes in iron transport. LCN2 induced T-cell apoptosis by reducing cellular iron concentration; moreover, the iron that was transported to the tumor microenvironment aided tumor cell proliferation, promoting tumor development. Meanwhile, LCN2 also influenced tumor progression through immune cytokines and cholesterol metabolism. Our results demonstrated that LCN2 has immunosuppressive functions that can promote tumor development; therefore, it is a potential immunotherapy target for CRC.

## Highlights

• Quantitative proteomics of tissue-infiltrating T cells in colorectal cancer patients.
• LCN2 was significantly increased in tumor-infiltrating T cells of colorectal cancer.
• LCN2 regulated T-cell iron efflux to decrease cellular iron and induced apoptosis.
• T-cell LCN2 promoted tumor cell proliferation *via* tumor microenvironment.

## Graphical Abstract

**Figure:**
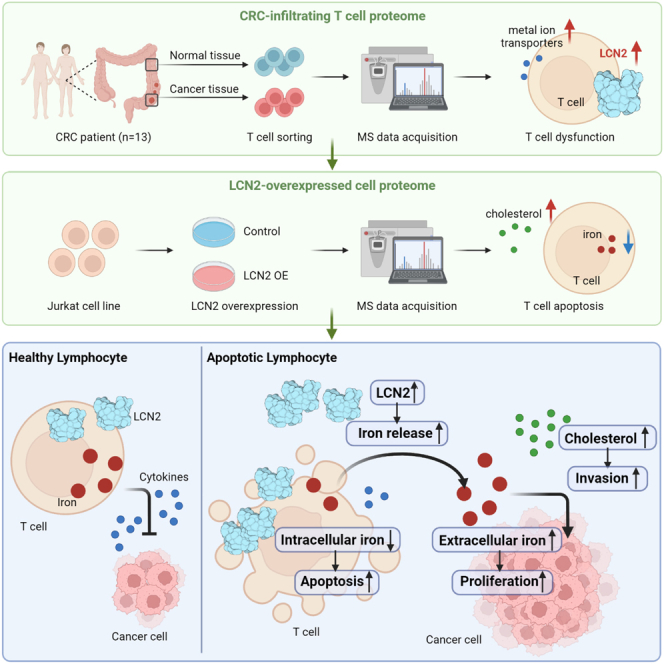

## In Brief

Che *et al.* described the protein expression pattern of tumor-infiltrating T cells in colorectal cancer using quantitative proteomic methods and screened a potential new immunotherapy target lipocalin-2 (LCN2). Proteomic analysis of LCN2-overexpressed Jurkat cells revealed that LCN2 reduced T-cell iron by regulating iron efflux, which induced T-cell apoptosis and promoted tumor cell proliferation *via* tumor microenvironment.

Proteins in the tumor immune microenvironment play key roles in tumor immunotherapy. In addition to tumor cells, the tumor microenvironment contains a variety of immune cells, stromal cells, cellular secretory substances, extracellular matrix (ECM) components, and blood and lymphatic vascular networks (1, 2). T cells are the most abundant immune cells in this microenvironment (3). Cancer-immunity cycle (CIC) refers to a series of immune responses that occur when T cells kill tumor cells; since T cells are the most pivotal participants in CIC, they are central to antitumor immunity (4, 5). Normally, T cells in tumors show abnormal protein expression patterns, resulting in T-cell dysfunction and a disrupted CIC, leading to an inability to kill tumor cells, and therefore enabling tumor development. As a result, describing T-cell protein expression patterns will help to understand the tumor immune microenvironment and antitumor immunity and to screen differentially expressed T-cell proteins, which may serve as potential new immunotherapy targets.

Colorectal cancer (CRC) is a malignant tumor lacking effective therapy. In 2020, there were approximately 1.9 million new CRC cases and 916,000 deaths worldwide (6); as a result, CRC ranked third in incidence and second in mortality of malignant tumors. However, there is currently no effective targeted therapy for CRC. For instance, immunotherapy treatment with anti-PD1 antibody was effective in only 4 to 5% of microsatellite instability-high tumors, which account for less than 15% of all CRC cases (7, 8). Therefore, immunotherapy for CRC has great development potential, but a comprehensive understanding of the CRC tumor immune microenvironment remains unknown. Therefore, CRC is a suitable disease model to perform proteomic profiling of tumor-infiltrating T cells.

Our study found that lipocalin-2 (LCN2) was significantly upregulated in tumor-infiltrating CD4^+^ T cells in CRC. LCN2, also known as neutrophil gelatinase–associated lipocalin, was first identified in 1993; it is a secretory protein that can regulate immunity and tumor progression (9). LCN2 is a 25 kDa protein with a bucket-like structure, which allows it to transport iron or other molecules in its pocket (10). As an iron transporter, LCN2 plays a bacteriostatic role by isolating iron. Depletion of LCN2 in mice leads to persistent bacteria colonization (11). Since iron is critical to cellular homeostasis, LCN2-mediated iron regulation can induce inflammation or even cell death. Increased LCN2 in retinal pigmented epithelial cells decreases autophagy and activates inflammasome-ferroptosis processes (12). In addition, LCN2 regulates immunity *via* cytokines. Increased LCN2 may enhance interleukins (IL-6 and IL-10) to induce inflammatory bowel diseases (13). Upregulated LCN2 in nephritic mice promotes IFN-γ and enhances inflammation, and the anti-LCN2 antibody can effectively alleviate nephritis (14). LCN2 is expressed in healthy tissues, including the kidney, liver, thymus, and small intestine. It is also expressed in cancerous colorectum, ovary, and spleen (15). Pan-cancer studies found that LCN2 expression is increased in lung cancer, breast cancer, and other tumors (16). LCN2 promotes tumor development by affecting cell proliferation, apoptosis, and migration (17). Upregulated LCN2 in tumor cells within cerebrospinal fluid allowed these cells to collect limiting iron to help their growth (18). Increased LCN2 in lung cancer promotes iron efflux, thereby avoiding iron accumulation and ferroptosis (19). Furthermore, LCN2 promotes tumor metastasis through the epithelial–mesenchymal transition in breast and prostate cancers (20, 21). However, most studies of LCN2 function in tumors have been conducted in tumor cells rather than immune cells; therefore, the LCN2 expression pattern and functions in tumor-infiltrating T cells remain unclear. This study fills in these blanks to expand our understanding of the functions of LCN2 in the tumor immune microenvironment.

In this study, we achieved proteomic analysis of tumor-infiltrating T cells in CRC by isotope labeling. We identified 7310 proteins, and 258 of these proteins were differentially expressed in CRC. We used bioinformatic analysis to describe the T-cell protein expression map and found that the antitumor immunity of T cells was suppressed. Accordingly, LCN2 was screened as a key differentially expressed protein (DEP); moreover, upregulated LCN2 in tumor-infiltrating T cells had an immunosuppressive function and could promote tumor development. Therefore, LCN2 is a potential immunotherapy target for CRC.

## Experimental Procedures

### Experimental Design and Statistical Rationale

To achieve protein profiling of CRC-infiltrating T cells and discovery of potential immunotherapy targets, tumor tissues and paired distant normal tissues (DNTs) (paracancerous tissues, 3 cm away from tumor tissues) from treatment-naïve CRC patients were collected, and T cells were sorted from the tissues using immunomagnetic beads, including CD4^+^ T cells and CD8^+^ T cells. A total of 46 T-cell samples were sorted from 13 CRC patients, including 26 CD4^+^ T-cell samples from paired tissues of 13 CRC patients and 20 CD8^+^ T-cell samples from paired tissues of 10 CRC patients. After five sets of 10-plex tandem mass tag (TMT) tag labeling (Supplemental Table S1), the fold changes (tumor/normal) of protein abundance were consistent with a normal distribution, which satisfied the prerequisite of paired *t* test. Paired *t* tests are suitable for analyzing paired datasets, such as T-cell protein abundances of tumor tissue and paired DNT from the same patient. DEPs in CRC were identified using paired *t* test (*p* < 0.05) and cutoff ratio (tumor/normal) >1.5 or <0.67. LCN2 expression levels were significantly increased in CRC-infiltrating T cells. LCN2-overexpressed Jurkat cell line was constructed, and quantitative proteomic analysis was performed to further explore LCN2 functions in T cells. Wildtype Jurkat cells were used as controls. A total of five paired cell samples were labeled using 10-plex TMT tags, including two paired samples without phytohemagglutinin (PHA) stimulation and three paired samples with PHA stimulation (Supplemental Table S2). The fold changes (LCN2-overexpressed/control) of protein abundance were consistent with a normal distribution as well. As these were not paired datasets, the DEPs in LCN2-overexpressed Jurkat cells were identified using edgeR (*p* < 0.05) and cutoff ratio (LCN2-overexpressed/control) >1.25 or <0.8. DEPs in CRC patients were used to screen potential immunotherapy targets, and the screening threshold is more stringent. DEPs in LCN2-overexpressed Jurkat cells were used to fully reveal the functions of LCN2, and the cutoff ratio was set differentially. LCN2 functions were explored in LCN2-overexpressed cell model and coculture systems.

### Patients and T-Cell Samples

Human colorectal specimens were obtained from Beijing Chao-Yang Hospital, Capital Medical University, Beijing, China, which were reviewed and approved by the Ethics Commission of Beijing Chao-Yang Hospital (2021-3-1-17). The patients/participants provided their written informed consent to participate in this study. All the procedures followed the Declaration of Helsinki principles. Patient clinical information after deidentification is summarized in Supplemental Table S3. After resection operation, tumor tissues and DNTs were used in hospital for clinicopathological detection, and the remaining tissues were collected, stored in transport buffer, and sent to Peking University by cold chain transport. Fresh tissues were washed with PBS to remove the remaining blood and transport buffer. The tissues were then cut and dissociated into single cell suspension using the Tumor Dissociation Kit (130-095-929; Miltenyi Biotec). Single cells were resuspended with sorting buffer and incubated with magnetic microbeads conjugated to monoclonal antihuman CD4 or CD8 antibodies (130-045-101 or 130-045-201; Miltenyi Biotec) for positive selection. Sorted T cells were kept frozen for subsequent proteomic study.

### Cell Lines, Cell Culture, and Treatments

All cell lines used in this study were obtained from American Type Culture Collection. Adherent cell lines 293T and HCT116 were cultured in Dulbecco's modified Eagle's medium supplemented with 10% fetal bovine serum (FBS); suspended cell lines Jurkat, K562, and Molt16 were cultured in RPMI1640 medium supplemented with 10% FBS; and all cell lines were cultured in humidified incubator at 37 °C with 5% CO_2_. For stable cell lines, overexpression plasmids were constructed by the T4 ligase system into a pLenti-CMV vector and transfected into 293T cells to acquire the lentivirus using Lipofectamine 3000 Transfection Reagent (L3000001; Invitrogen); stable cell lines were infected with the lentivirus and selected by treatment with 10 μg/ml BSD (461120; Invitrogen). Cell lines with fluorescent tags were directly sorted using flow cytometry. Stable cell lines were validated by Western blotting assay. Jurkat cells were stimulated and activated with 2 μg/ml PHA (11249738001; Roche). Cells were treated with deferoxamine (DFO) (ab120727; Abcam), ferric citrate (FC) (F3388; Sigma–Aldrich), low-density lipoprotein-cholesterol (LDL-C) (H7960; Solarbio), or total cholesterol (S4154; Selleck Chemicals) as indicated.

### Protein Extraction and Digestion

Sorted T cells were lysed with 8 M urea, and protein concentration was determined using protein quantification assay (23227; Thermo Fisher Scientific). Approximately 100 μg protein was reduced with 5 mM dithiothreitol for 30 min at room temperature and then alkylated with 10 mM iodoacetamide in the dark for 30 min. The protein was digested into peptides by Lys-C (125-05061; Wako Chemicals) for 3 h and trypsin (V5111; Promega) overnight at 37 °C. The digestion was terminated by 0.5% aqueous TFA.

### TMT Labeling

Digested peptides were desalted, dried in vacuum, and resuspended in 100 mM tetraethylammonium bromide, and peptide concentration was determined accurately using protein quantification assay. A total of five sets of 10-plex TMT reagents (90110; Thermo Fisher Scientific) were used to label 46 T-cell samples from CRC patients, and a set of 10-plex TMT reagent was used to label 10 Jurkat cell samples. TMT reagent was dissolved in 41 μl acetonitrile, and 20 μg peptide was labeled with 10 μl TMT reagent for 1 h at room temperature. The labeling was terminated by 5% aqueous hydroxylamine for 20 min. Each set of TMT-labeled samples were mixed into one mixture.

### Peptide Fractionation

TMT-labeled peptides were dried in vacuum and resuspended in ultrapure water (pH = 10) and were fractionated using a tip fractionation column containing C18. Peptides were eluted with gradient buffer of aqueous acetonitrile (10%, 12.5%, 15%, 17.5%, 20%, 22.5%, 25%, and 50%) in volume of 200 μl. Elution peptides with 10% and 50% aqueous acetonitrile buffer were mixed into one mixture, resulting in seven fractions for each set of TMT-labeled peptides. The fractions were dried in vacuum and stored at −80 °C until LC–MS/MS analysis.

### LC–MS/MS Analysis

The dried peptides were resuspended in 20 μl 0.2% aqueous formic acid. After centrifugation at 12,000*g* for 5 min, 10 μl supernatant was used for LC–MS/MS analysis. A Nano EASY-nLC 1200 HPLC system (Thermo Fisher Scientific) coupled to an Orbitrap Fusion Lumos Tribrid Mass Spectrometer (Thermo Fisher Scientific) was used to perform LC–MS/MS analysis. The peptides were loaded on a 75 μm × 2 cm, 3 μm Acclaim PepMap NanoViper C18 trap column with 0.1% aqueous formic acid at 8 μl/min flow rate and analyzed using a 75 μm × 25 cm, 2 μm PepMap RSLC C18 analytical column with acetonitrile gradient 6 to 90% buffer (0.1% formic acid in 80% aqueous acetonitrile) at 300 nl/min flow rate. Mass spectrometric (MS) data were collected using data-dependent acquisition. The main parameters of MS were set as follows: method duration of 194 min, ion source type was nanospray ion source, spray voltage of positive ion and negative ion was set at 2200 V and 600 V, respectively, ion transfer tube temperature of 320 °C, and default charge state of 2. Precursor ions (MS1) scanned were further dissociated by high-energy collisions at a normalized collision energy setting of 37%, and produced fragment ions (MS2) were further detected. Orbitrap resolution was set to 120,000 and 50,000 for MS1 and MS2. Maximum injection time was set to 100 ms and 86 ms for MS1 and MS2. Automatic gain control target was set to 1,000,000 and 100,000 for MS1 and MS2. Scan range *m/z* was of 300 to 1500 and RF lens of 30%.

### MS Database Searching

Raw data were analyzed using Proteome Discoverer software (version 2.2.0.388; Thermo Fisher Scientific) with search engine SEQUESH. Databases used to identify protein were downloaded from UniProt *Homo sapiens* database. The database for proteomics of clinical samples was downloaded on September 8, 2017, containing 93,274 entries, and the database for proteomics of LCN2-overexpressed cell line was downloaded on February 26, 2019, containing 95,556 entries. Searches were configured with two kinds of fixed modifications, including TMT reagent modification on lysine and any N terminus (+229.163 Da) and carbamidomethyl on cysteines (+57.021 Da) and two kinds of variable modifications, including oxidation on methionine residues (+15.995 Da) and acetyl on N terminus (+42.011 Da). Peptides were generated using Lys-C and trypsin, permitting two missed cleavage sites, the maximum peptide length of 144, and the minimum peptide length of 6. Mass tolerance for precursor ions was 10 ppm, and mass tolerance for fragment ions was 0.02 Da. The false discovery rate was calculated by Percolator algorithm in Proteome Discoverer Software, false discovery rate at peptide and protein level of 1%, and at least one peptide segment of the protein was identified as specific to this protein. Normalization based on total reporter ion intensity was used to balance channel errors. Normalized protein abundances were used for further analysis.

### RT–PCR

Total RNA was obtained from cells using the Ultrapure RNA Kit (CW0597; CoWin Biosciences) according to the manufacturer’s instructions. Next, 4 μg RNA was reverse transcribed into complementary DNA (cDNA) using the HiFiScript cDNA Synthesis Kit (CW2569; CoWin Biosciences) in a 20 μl reaction volume. cDNA was amplified with the GoTaq qPCR Master Mix (A6001; Promega) and analyzed using the CFX96 Touch Real-Time PCR System (Bio-Rad). RT–PCR results were quantified using Bio-Rad CFX Manager software (version 3.1) with GAPDH as the internal control. All primers were purchased from Tsingke, and sequences are provided in Supplemental Table S4.

### Western Blotting

Cells were lysed by 1% aqueous SDS with an ultrasonic processor. After protein quantification assay, samples containing 20 μg protein were denatured, separated by SDS-PAGE, and transferred onto polyvinylidene fluoride membranes (1620177; Bio-Rad). Next, the membranes were blocked with 5% skim milk in Tris-buffered saline with Tween (TBST) and subsequently incubated with primary antibodies against GAPDH (G8795; Sigma–Aldrich), β-actin (ab6276; Abcam), chromogranin-A (CHGA; 60135; Proteintech), calponin-2 (CNN2; 21073; Proteintech), ferritin light chain (FTL; 10727; Proteintech), hypoxia-inducible factor 1-alpha (20960; Proteintech), caspase-3 (9662; CST), caspase-7 (9492; CST), FPN1 (solute carrier family 40 member 1; 106732; Solarbio), LCN2 (A2092; ABclonal), ACAT2 (acetyl-CoA acetyltransferase; A1399; ABclonal), HMGCS1 (hydroxymethylglutaryl-CoA synthase, cytoplasmic; A3916; ABclonal), mevalonate kinase (MVK; A20906; ABclonal), MVD (diphosphomevalonate decarboxylase; A0813; ABclonal), LSS (lanosterol synthase; A6930; ABclonal), or SREBP2 (sterol regulatory element–binding protein 2; A13049; ABclonal) overnight at 4 °C. The membranes were washed with TBST and incubated with secondary antimouse (1030-05; SouthernBiotech) or anti-rabbit (4010-05; SouthernBiotech) antibodies for 1 h at room temperature. Finally, the membranes were washed with TBST and detected with the ECL Kit (WBULS0500; Millipore).

### Flow Cytometry

After cell counting, cells were washed with PBS and stained with antibodies from BD Biosciences (Franklin Lakes) against CD3-FITC (555916), CD4-PE (561843), and CD8-PE (561949). For the cell apoptosis assay, cells were stained with the Apoptosis Detection Kit (AD10; Dojindo) according to the manufacturer’s instructions, and annexin V was measured by fluorescent signal FITC. The cells were then washed again with PBS and resuspended with 2% FBS in PBS. The cell samples were injected into the BD FACSVerse Cell Analyzer (BD Biosciences), and the flow cytometry standard data were analyzed using FlowJo software (version 10.0.7; BD Biosciences).

### Immunofluorescence

For immunofluorescence, the cells were fixed with 4% aqueous paraformaldehyde and treated with 0.5% Triton X-100 in PBS. Cells were washed with PBS after each treatment. After blocking with 5% bovine serum albumin in PBS for 1 h at room temperature, the cells were incubated with primary antibodies against LCN2 (A2092; ABclonal) overnight at 4 °C. The cells were then incubated with fluorescent secondary anti-rabbit antibodies (A11034; Invitrogen) for 1 h at room temperature in the dark. The cells were washed with PBS, stained with 4′,6-diamidino-2-phenylindole (C1005; Beyotime) for 10 min at room temperature and then photographed using the LSM 880 confocal microscope (Carl Zeiss).

### Cell Proliferation Assay

Cell proliferation was measured by Cell Counting Kit-8 (CCK-8) assay and carboxyfluorescein succinimidyl ester (CFSE) staining. For the CCK-8 assay, cells were seeded into 96-well plates and incubated with the indicated treatments. Briefly, 100 μl of fresh medium with 10 μl of CCK-8 solution (C0041; Beyotime) was added to the cells and incubated at 37 °C with 5% CO_2_ for 2 h. Absorbance was measured at 450 nm using a microplate reader. For the CFSE-based cell proliferation assay, cells were incubated with 10 μM CFSE (565082; BD Biosciences) in an incubator for 15 min. Subsequently, cells were washed and cultured with indicated treatments, and CFSE signals were detected by flow cytometry.

### Iron Detection

Cellular iron was measured by calcein-AM assay. The cells were stained with 0.5 μM iron-sensitive calcein-AM solution (40719ES50; Yeasen) in an incubator at 37 °C with 5% CO_2_ for 30 min. The cells were then washed, and fluorescent signals were detected by flow cytometry. Iron in the cell supernatant was measured using inductively coupled plasma optical emission spectrometry (ICP-OES), which was performed by The College of Environmental Sciences and Engineering, Peking University, Beijing, China.

### Cholesterol Detection

For cholesterol detection, the cells and supernatant were separated by centrifugation at 800*g* for 2 min. To disrupt the cells, they were washed with PBS, frozen, and melted for three cycles. Cell samples were collected by centrifugation at 1500*g* for 10 min. Supernatant samples were collected by further centrifugation at 3000*g* for 20 min to remove impurities. Total cholesterol in cells or cell supernatants was measured using the Human Total Cholesterol ELISA Kit (F10387-A; Fankewei) according to the manufacturer’s instructions.

### Reactive Oxygen Species Detection

Reactive oxygen species (ROS) was measured using the ROS Assay Kit (S0033; Beyotime). First, the cells were stained with 2'-7'dichlorofluorescin diacetate probes in an incubator at 37 °C with 5% CO_2_ for 20 min and then washed with and resuspended in PBS. ROS was measured by fluorescent signal FITC using flow cytometry, or 10^5^ cells were seeded into 96-well plates and measured using fluorescence microplate reader at 488 nm.

### Colony Formation

For the colony formation assay, 10^4^ cells were seeded into 6-well plates and incubated with the indicated treatments. After 7 days of cell proliferation, the cells were fixed with 4% aqueous paraformaldehyde for 15 min and stained with crystal violet dye (C0121; Beyotime) for 20 min. Subsequently, the cells were washed with PBS and photographed under a microscope. A minimum of five images were photographed.

### Cell Invasion Assay

Transwell insert chambers with 8 μm porous membranes (Corning) were used for the cell invasion assay. Matrigel (354248; Corning) was diluted with FBS-free medium to 200 μg/ml, and 100 μl of the diluted Matrigel was added to the top chamber and placed in an incubator at 37 °C for 1 h. HCT116 cells with indicated treatments in FBS-free medium were placed in the top chamber at a density of 1.5 × 10^4^ cells per well, complete medium was added to the bottom chamber, and the cells were incubated for 24 h. To quantify the invasive cells, noninvasive cells on the upper side of the membranes were removed. Invasive cells on the lower side were fixed with 4% aqueous paraformaldehyde for 15 min and stained with crystal violet dye (C0121; Beyotime) for 20 min. Subsequently, the cells were washed with PBS and photographed under a microscope. A minimum of five images were photographed.

### Cell Coculture Systems

HCT116 cells and Jurkat cells were cultured in a 1:1 ratio for 48 h in an incubator at 37 °C with 5% CO_2_. Transwell insert chambers with 4 μm porous membranes (Corning) were used for noncontact coculture; Jurkat cells were added to the top chamber, and HCT116 cells were added to the bottom chamber. The cell counting assay of HCT116 cells was performed using CCK-8 after coculturing. For direct coculture, HCT116-enhanced GFP and Jurkat-mCherry cell lines were constructed; after coculturing, the cells were separated by flow cytometry for further use.

### Bioinformatic Analysis and Statistical Analysis

RStudio software (version 1.4.1106) was used for bioinformati analysis. DEPs in CRC patient samples and LCN2-overexpressed Jurkat cells were screened by paired *t* test and the R package edgeR, respectively. Volcano plots were drawn using the R package ggplot2, and heatmaps were drawn using the R package pheatmap. Cellular component distribution of identified proteins was performed using FunRich software (version 3.1; http://www.funrich.org/). STRING database (version 11.5; https://string-db.org/) was used for enrichment analysis, including Gene Ontology (GO) enrichment, Reactome pathway enrichment, and protein–protein interaction (PPI) network analysis. Moreover, the interaction intensity scores in the PPI network were evaluated using Cytoscape software (version 3.9.1; https://cytoscape.org/). For validation of DEPs at the RNA level, the GEPIA (Gene Expression Profiling Interactive Analysis) database (http://gepia.cancer-pku.cn/) was used.

GraphPad Prism software (version 6.01; GraphPad Software, Inc) was used to perform statistical analysis. All measurement values were expressed as means ± SEM. Each experiment was performed at least three times (independent experiments using three technical replicates). Statistical comparisons between different treatments were performed using the unpaired Student’s *t* test, and results were considered significant at *p* < 0.05. Significance thresholds were defined as ∗*p* < 0.05, ∗∗*p* < 0.01, and ∗∗∗*p* < 0.001.

## Results

### A Relative Quantitative Proteomic Study of T Cells in CRC Patient Tissues

**Fig. 1:**
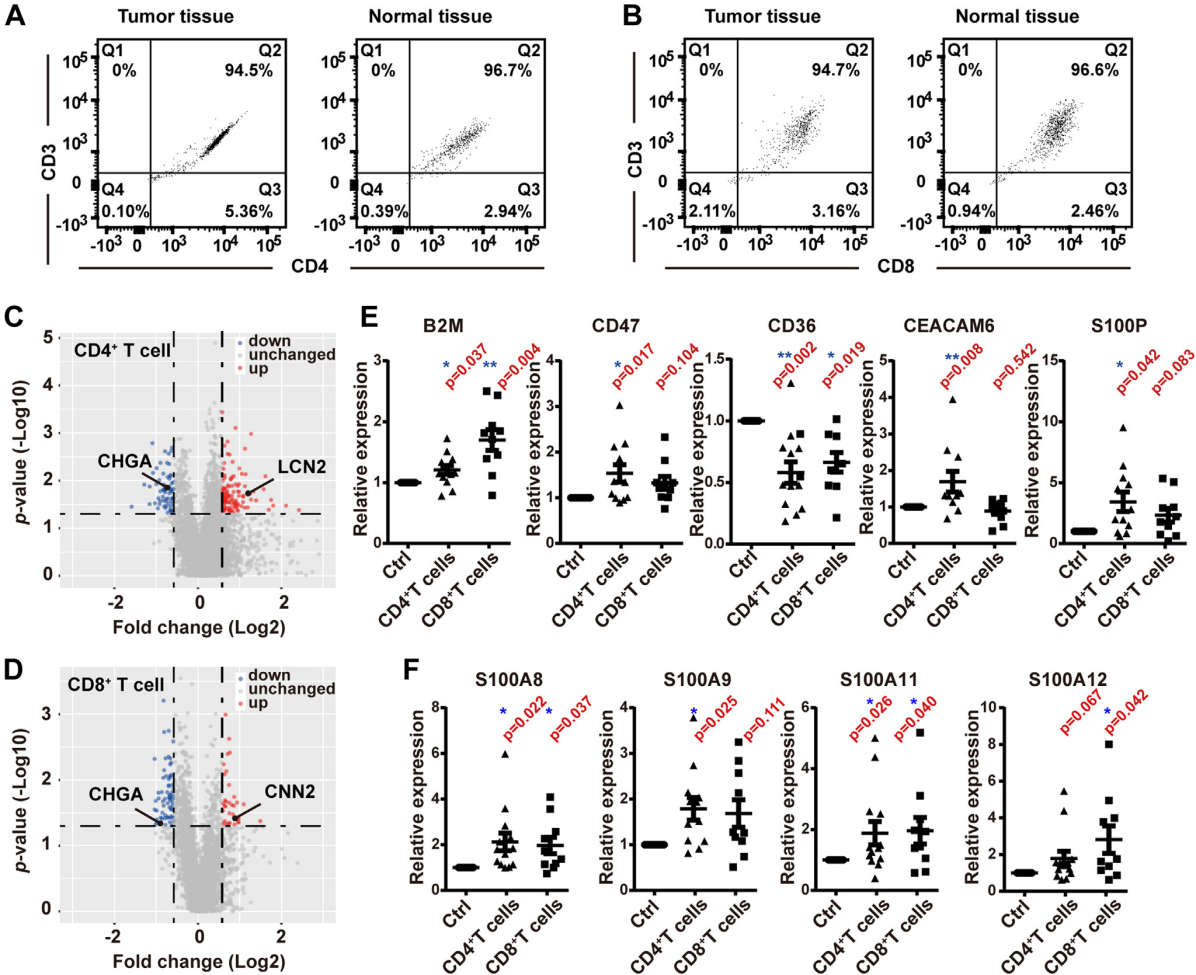
**Validation of the purity of sorted T cells and DEPs screening from CRC patient proteome data.** The purity of sorted (*A*) CD4^+^ T cells and (*B*) CD8^+^ T cells was confirmed using flow cytometry. DEPs from (*C*) CD4^+^ T cells and (*D*) CD8^+^ T cells were visualized using volcano plots, with significantly upregulated proteins (fold change >1.50, *p* < 0.05) in *red* and downregulated proteins (fold change <0.67, *p* < 0.05) in *blue*. *E*, known immunotherapy targets, clinical diagnostic markers, and (*F*) potential diagnostic markers among these DEPs. Statistical analysis was performed using a paired *t* test of tumor samples to normal samples. ∗*p* < 0.05, ∗∗*p* < 0.01. CRC, differentially expressed protein; DEP, differentially expressed protein.

To describe the characteristics of T cells in CRC, we performed a relative quantitative proteomic study of infiltrating T cells in CRC clinical samples. We collected tumor tissues and paired DNTs from 13 male and female CRC patients, ranging from stage I to III. Both CD4^+^ and CD8^+^ T cells were isolated from the tissues by an immunomagnetic bead–based magnetic-activated cell sorting separation system, resulting in 10^5^ cells obtained from 0.5 g fresh tissue. Because of tissue size limitations, CD4^+^ T cells were successfully sorted from all 13 patients, but CD8^+^ T cells were sorted from 10 patients (patient #1–13 and patient #1–10, respectively; Supplemental Table S3). Using flow cytometry against CD3-FITC, CD4-PE, and CD8-PE, the purity of the sorted cells was found to be approximately 95% (Fig. 1, *A* and *B*), ensuring the validity of subsequent proteomic studies. We then performed a relative quantitative proteomic study of the sorted T cells by TMT labeling. Among the 7310 identified proteins, 6952 CD4^+^ T-cell proteins and 6298 CD8^+^ T-cell proteins had quantification information, with 6194 proteins in common between these two types of T cells (Supplemental Table S5).

Next, we screened DEPs to understand changes in T cells in CRC. We identified 258 DEPs by paired *t* test (fold change >1.5 or <0.67; *p* < 0.05) in CRC pairwise, among which 115 proteins were upregulated and 66 proteins were downregulated in CD4^+^ T cells (Fig. 1*C*); and 35 proteins were upregulated and 64 proteins were downregulated in CD8^+^ T cells (Fig. 1*D*). The DEP list is shown in Supplemental Table S6. Several known immunotherapy targets and clinical diagnostic markers were screened (Fig. 1*E*). Increased B2M in glioma immune cells is positively correlated with the expression of immune checkpoints such as PD-L1 and TIM-3 (22). B2M is a potential therapy target and can be used to predict poor prognosis in glioma (23, 24). The classical immunotherapy target CD47 is enhanced in many solid tumors (25); furthermore, the US Clinical Trials Registry (ClinicalTrials.gov) showed a total of 23 CD47-targeting drugs with 46 clinical trials as of August 2021 (26). CD36 is upregulated in regulatory T cells in breast cancer and melanoma but tends to be downregulated in CRC (27, 28). Reports have shown that decreased CD36 in CRC is related to poor prognosis (29). Carcinoma embryonic antigen (CEA) family members serve as clinical diagnostic markers (30). CEACAM6 is particularly applicable in myeloid leukemia and colon adenoma (31), and therapeutic antibodies against CEACAM6 have also been tested in clinical trials (32). Moreover, the diagnostic marker S100P is significantly enhanced in almost all tumors (33, 34). Other members of the S100 family, such as S100A8, S100A9 (35), S100A11 (36), and S100A12 (37) (Fig. 1*F*), have also demonstrated potential use as clinical markers. Altogether, these results supported the effectiveness of our screening method. This proteomic study accurately reflected the protein expression characteristics of tumor-infiltrating T cells. Accordingly, these DEPs could then be annotated to describe the protein expression pattern of T cells in CRC.

### Protein Expression Pattern of T Cells in CRC

**Fig. 2:**
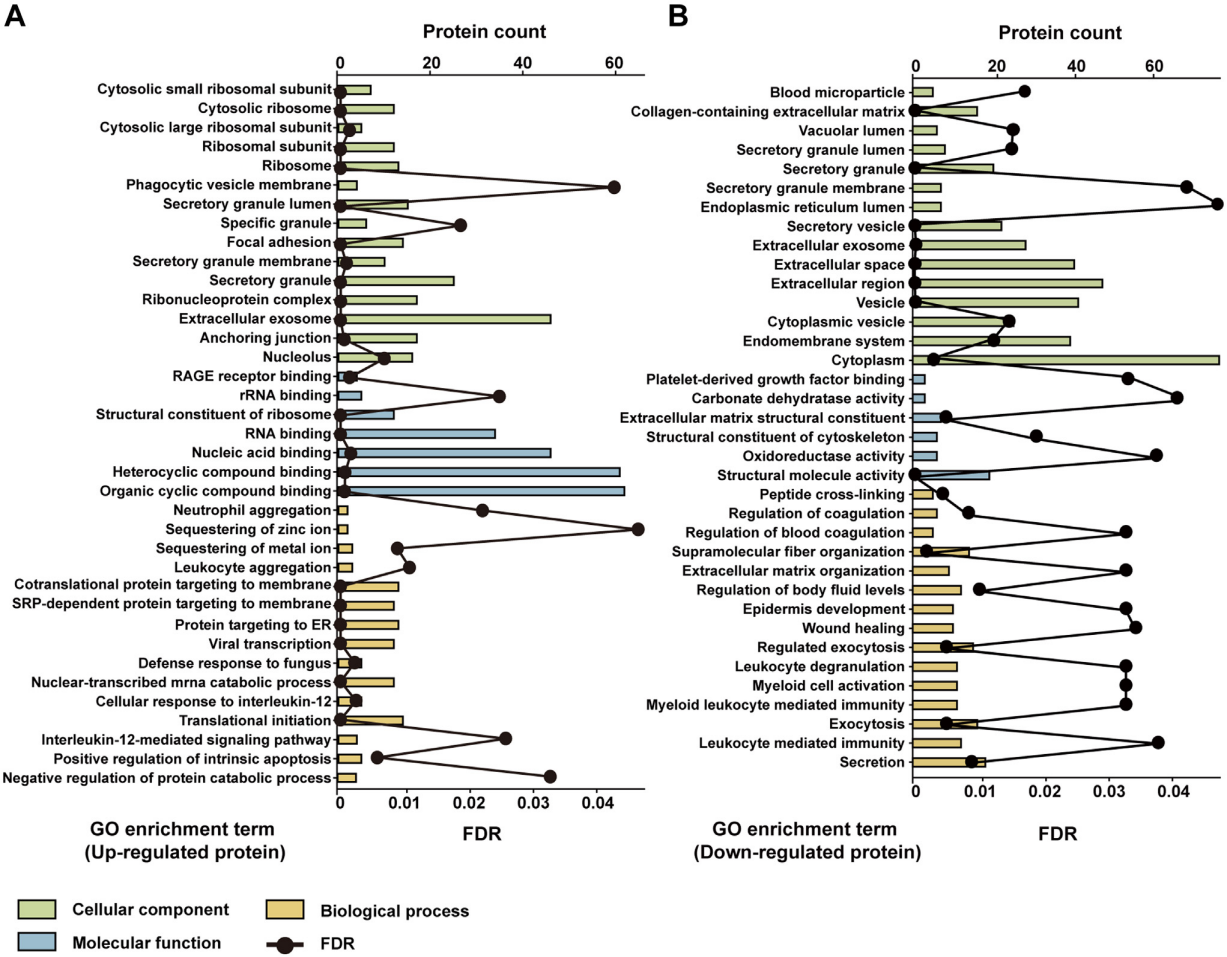
**Gene Ontology (GO) enrichment analysis of the DEPs from T cells in CRC patient tumors.** GO enrichment analysis of significantly (*A*) upregulated and (*B*) downregulated proteins. For the enrichment results with more than 15 terms, only the top 15 terms with the strongest enrichment intensity are shown here. CRC, colorectal cancer; DEP, differentially expressed protein.

**Supplemental Figure S1:**
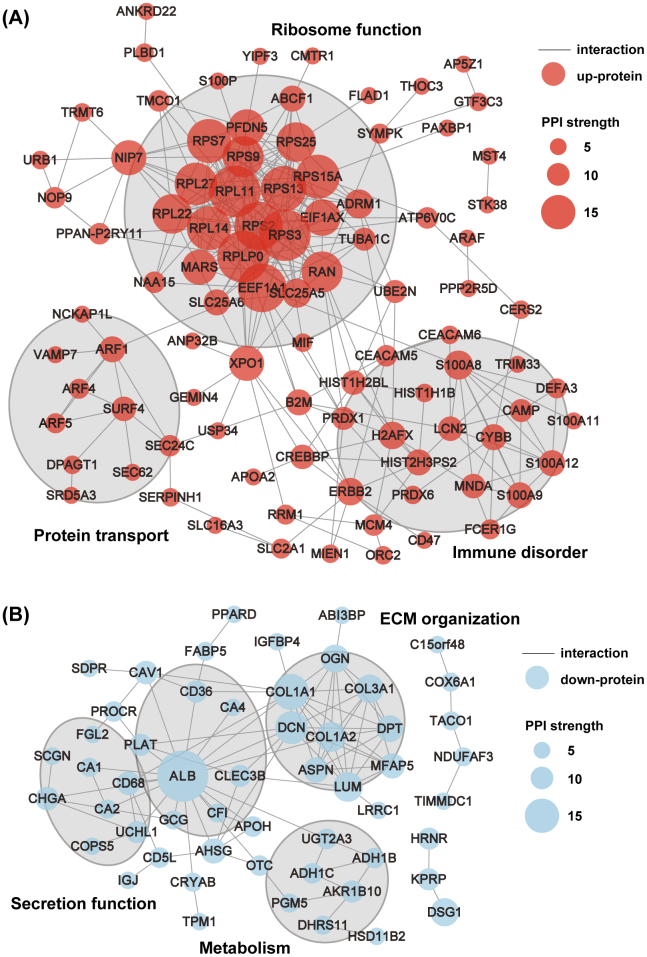


Next, we annotated the DEPs to describe the protein expression pattern of T cells in CRC. The STRING database was used to perform GO enrichment analysis of the DEPs, including cellular component, molecular function, and biological process. Most of the upregulated proteins were clustered in ribosome subunits and cell structures related to protein secretion, and their functions were primarily related to binding of RNA, DNA, or other compounds. Their biological process enrichment showed not only participation in cell homeostasis mediated by metal ion transporters but also involvement in immune processes (Fig. 2*A*). Downregulated proteins were primarily shown to be secretory proteins located in secretion-related cellular structures or the ECM, with molecular functions related to cytoskeleton maintenance and ECM organization. As for biological process, downregulated proteins were mainly enriched in cell structure maintenance, lymphocyte activation, and immune responses through lymphocytes (Fig. 2*B*). On this basis, PPI network analysis (Supplemental Fig. S1) was performed to reveal the proteins involved in these specific T-cell characteristics.

The enrichment results for the upregulated proteins revealed the following characteristics of T cells in CRC: active ribosomes and disturbance of cellular homeostasis with increased inflammation. First, eight S40 subunit proteins (RPS2, RPS3, RPS7, RPS9, RPS13, RPS15, RPS24, and RPS25) and five S50 subunit proteins (RPLP0, RPL11, RPL14, RPL22, and RPL27) were significantly increased in CRC. Tumor-induced reprogramming of the immune microenvironment dramatically increases the demand of protein synthesis, leading to active ribosomes. Moreover, an increased number of ribosomes and an upregulation of ribosomal proteins have been shown to be associated with tumor progression (38). Second, the cellular homeostasis of T cells was shown to be disturbed based on the enhancement of the metal ion transporters S100A8, S100A9, LCN2, and CNN2, which are pivotal for activity of metalloproteinases and may trigger acute cell stress, leading to T-cell damage. Furthermore, the upregulated S100 family members S100P, S100A8, S100A9, and S100A12 function as immunosuppressive factors by binding receptors for advanced glycation end products, thereby enhancing the expression of damage-associated molecular pattern molecules (39). In addition, these T cells exhibited strong inflammation characteristics, including immune cell recruitment, cytokine secretion, and cell stress. For example, secretion of macrophage migration inhibitory factor (MIF) was enhanced. MIF contributes to glucocorticoid-mediated immune suppression, and inhibitors targeting MIF are being studied in clinical trials (40). Increased MIF, CNN2, and ARF1 are enriched in IL-12-related pathways, which promote tumor development (41, 42, 43).

Enrichment of the downregulated proteins revealed two additional T-cell characteristics: cell infiltration and immune suppression. First, the collagens COL1A1, COL1A2, and COL3A1, and another ECM component DCN, were decreased in CRC, which relate to enhanced cell migration (44). Consistent with this finding, MMP2 was 1.49-fold upregulated in CD4^+^ T cells and 1.57-fold upregulated in CD8^+^ T cells, which promotes cell migration by degrading the ECM (45). Second, T cells contained less T-cell activation regulator FGL2 (46). In addition, the calcium ion regulator CHGA was downregulated, which regulates calcium ion flow to affect immune cell activation. Furthermore, the metabolism regulators CD36, FABP5, and DEGS1 were downregulated, which may suppress immunity *via* disrupted T-cell metabolism.

Collectively, these results described the T-cell protein expression pattern and revealed T-cell characteristics in CRC.

### LCN2 is a Key DEP for Further Exploration

**Fig. 3:**
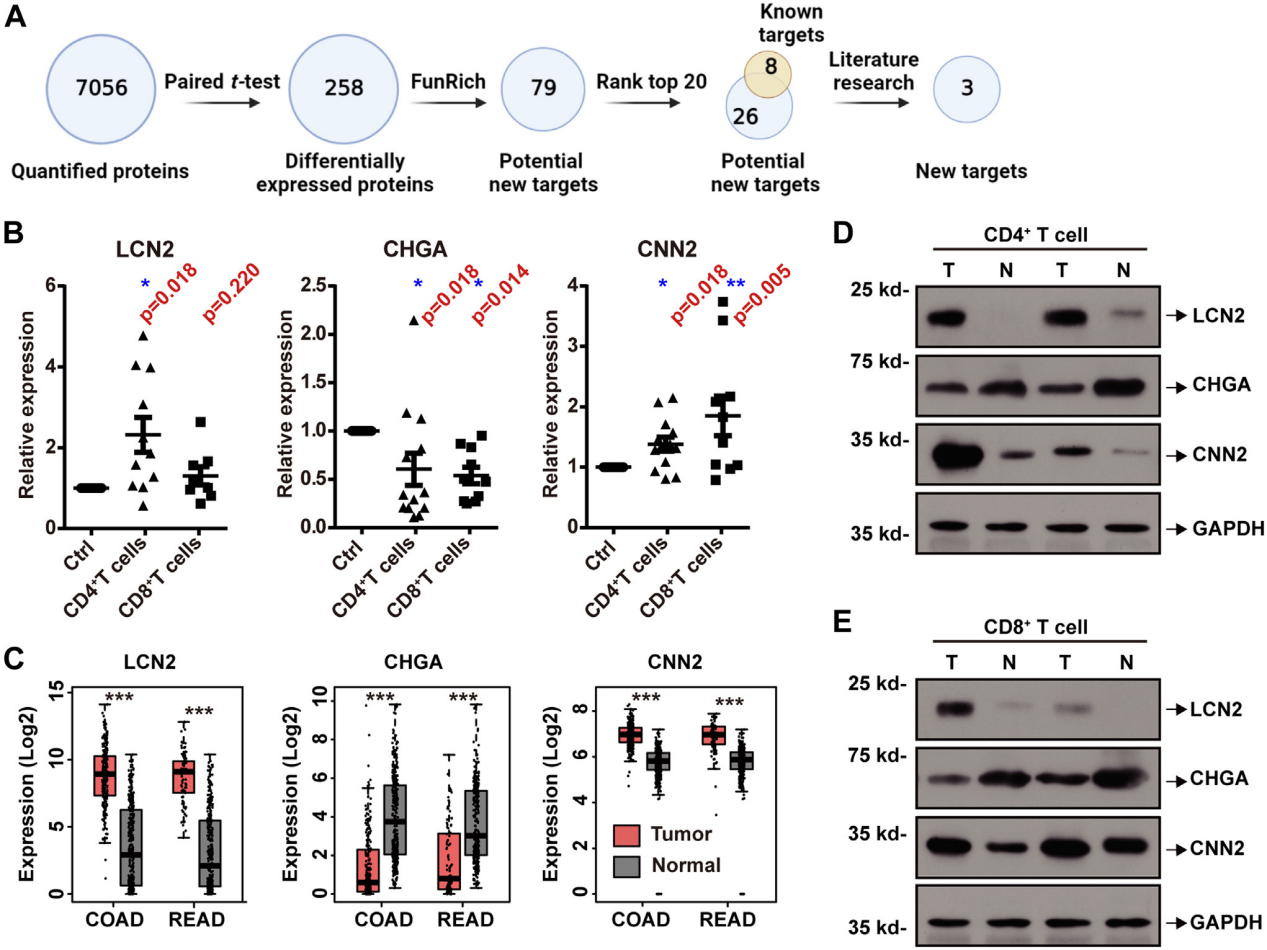
**Screening and validation of DEPs reveals that LCN2 is a key DEP in CRC tumor–infiltrating T cells.***A*, schematic of the workflow process used to screen key DEPs, created with biorender.com. *B*, LCN2, CHGA, and CNN2 were screened as a result of the workflow process in (*A*). These three key DEPs were validated (*C*) at the mRNA level using RT–PCR and (*D* and *E*) at the protein level using Western blotting. Statistical analysis was performed using a paired *t* test of tumor samples to normal samples. ∗*p* < 0.05, ∗∗*p* < 0.01, and ∗∗∗*p* < 0.001. COAD, colon adenocarcinoma; CRC, colorectal cancer; DEP, differentially expressed protein; READ, rectum adenocarcinoma.

To screen the most important DEP with the greatest potential for further exploration, multiple screening steps were used (Fig. 3*A*), and three key DEPs were screened: LCN2, CHGA, and CNN2 (Fig. 3*B*). Prior to further study, we validated their expression at the RNA level using the GEPIA database (Fig. 3*C*) and at the protein level by performing Western blotting of the two remaining clinical samples (Fig. 3, *D* and *E*; patient #14–15 in Supplemental Table S3). These results were consistent with the previous screening results.

We chose LCN2 for further exploration because of its larger fold change and better consistency across the different samples (Figs. 1, *C* and *D* and 3*B*). Although LCN2 has been confirmed to be upregulated in a variety of tumors, and has the potential to affect tumor progression by affecting processes such as cell proliferation, apoptosis, and migration, the functions of LCN2 in tumor-infiltrating T cells require clarification.

### LCN2 Promoted Lymphocyte Apoptosis

**Supplemental Figure S2:**
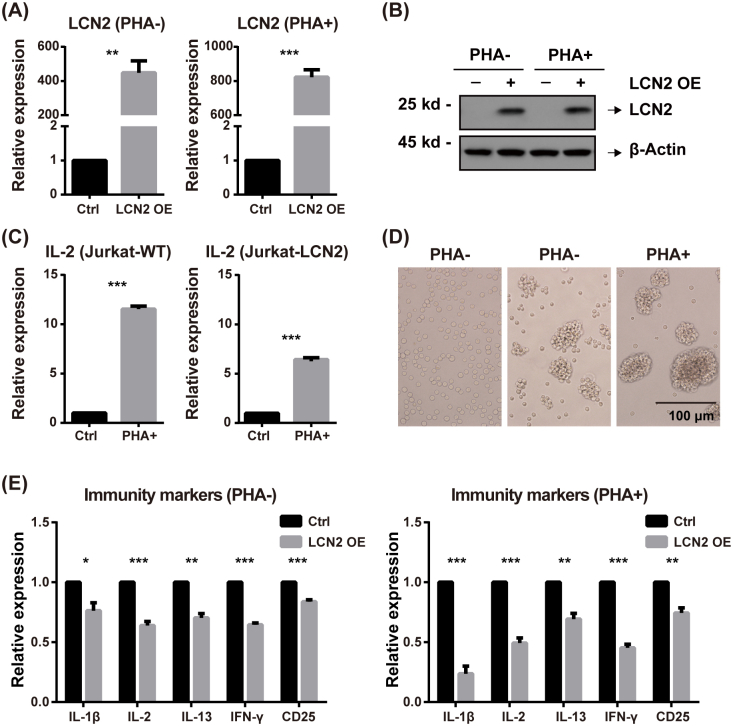


To explore the functions of LCN2 in T cells, a cell line overexpressing LCN2 was constructed using the acute T-cell leukemia cell line Jurkat. LCN2 overexpression was validated at both the mRNA (Supplemental Fig. S2*A*) and protein levels (Supplemental Fig. S2*B*), with or without PHA stimulation. To stimulate and activate the T cells, 2 mg/ml PHA was used, and cell activation was confirmed by IL-2 detection and morphological observation (Supplemental Fig. S2, *C* and *D*). Interestingly, PHA stimulation increased IL-2 levels 12-fold in wildtype Jurkat cells but only sixfold in LCN2-overexpressed Jurkat cells (Supplemental Fig. S2*C*), indicating the potential immunosuppressive function of LCN2. We further detected several important cytokines (IL-1β, IL-2, IL-13, and IFN-γ) and another T-cell activation marker CD25 in LCN2-overexpressed Jurkat cells; results suggested that LCN2 had immunosuppressive effects (Supplemental Fig. S2*E*).

**Fig. 4:**
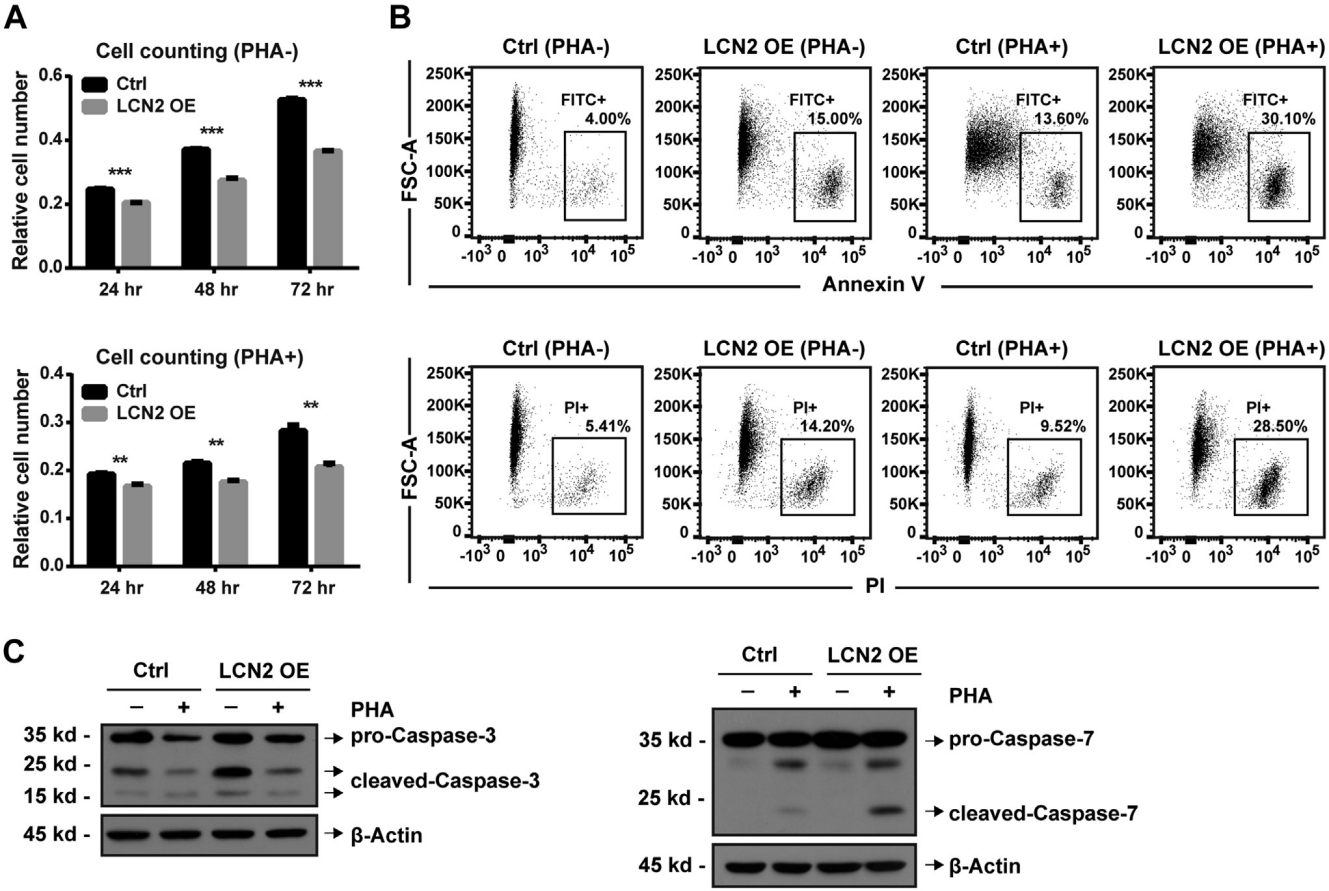
**LCN2 reduced Jurkat cell number by promoting cell apoptosis.** The CCK-8 assay was performed in (*A*) LCN2-overexpressed Jurkat cells (LCN2 OE) with or without PHA treatment. *B*, cell apoptosis was measured by annexin V and propidium iodide (PI) signals using flow cytometry. *C*, cell apoptosis markers caspase-3 and caspase-7 were measured at the protein level using Western blotting. Statistical analysis was performed using a *t* test from representative results of three similar experiments. ∗∗*p* < 0.01, ∗∗∗*p* < 0.001. CCK-8, Cell Counting Kit-8; PHA, phytohemagglutinin.

**Supplemental Figure S3:**
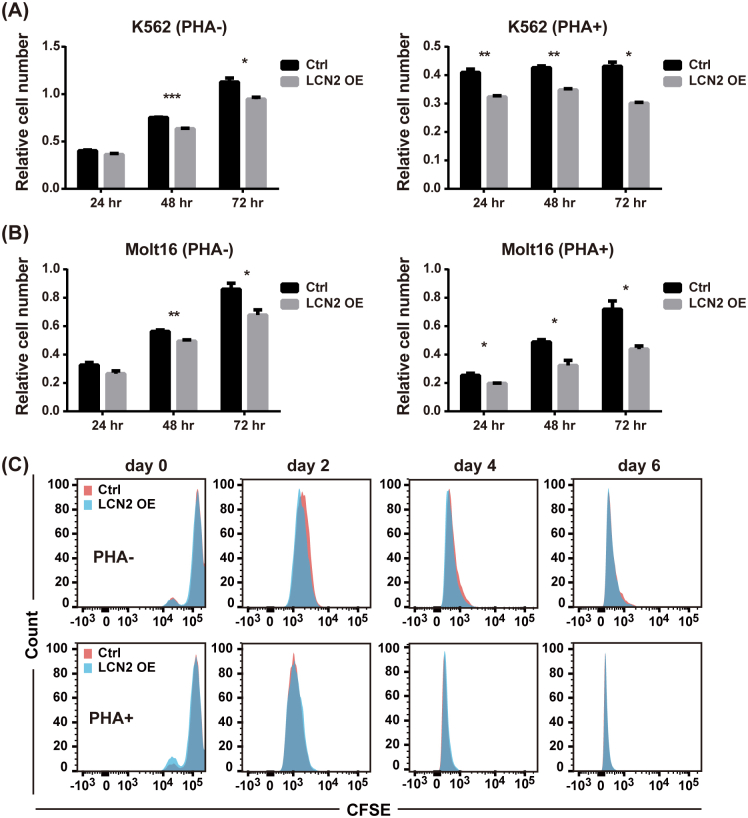


After LCN2 overexpression, the CCK-8 cell counting assay was performed, which showed that LCN2 overexpression resulted in a decreased cell number of Jurkat cells (Fig. 4*A*). This phenotype was further confirmed in the chronic myeloid leukemia cell line K562 (Supplemental Fig. S3*A*) and another acute T-cell leukemia cell line, Molt16 (Supplemental Fig. S3*B*). In order to clarify whether the decreased cell number was due to decreased cell proliferation or increased apoptosis, we measured cell proliferation using CFSE staining. LCN2 had no effect on cell proliferation (Supplemental Fig. S3*C*); therefore, we speculated that LCN2 promotes cell apoptosis. We used flow cytometry to detect the cell apoptosis signals annexin V/propidium iodide (PI) and found that annexin V and PI were increased by threefold in cells overexpressing LCN2 (Fig. 4*B*), demonstrating that LCN2 promoted cell apoptosis. This finding was further confirmed by performing Western blotting against caspase-3 and caspase-7, which showed increases in cleaved caspases in cells overexpressing LCN2 (Fig. 4*C*). Taken together, these results indicate that LCN2 promotes cell apoptosis.

### LCN2 Induced Lymphocyte Apoptosis by Reducing Cellular Iron

**Fig. 5:**
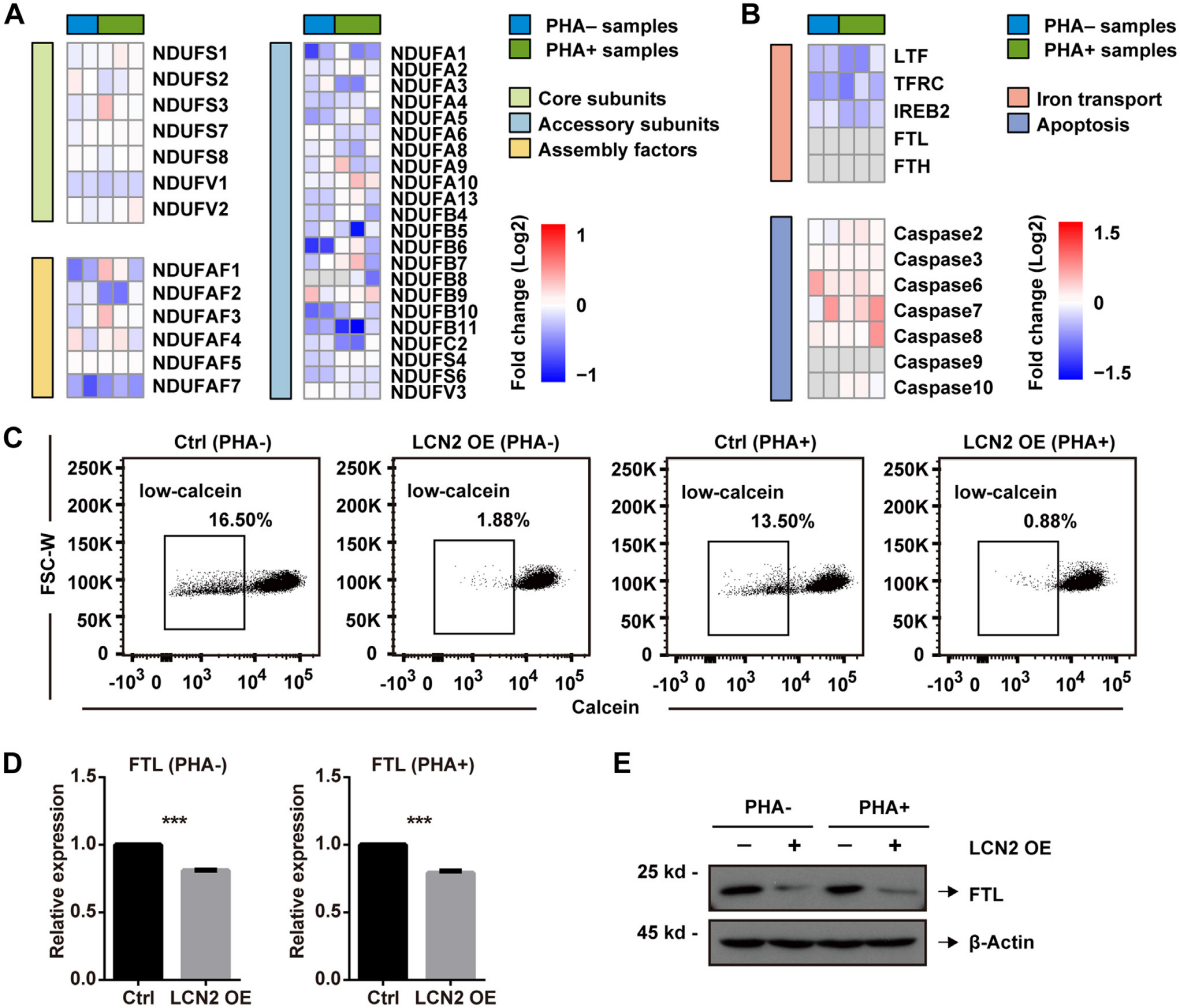
**LCN2 reduced cellular iron concentration in Jurkat cells.***A*, heatmaps of proteins related to complex I, including core subunits (*light green*), accessory subunits (*light blue*), assembly factors (*light yellow*), (*B*) and cellular iron concentration (*light red*) and cell apoptosis (*light blue*). *C*, cellular iron concentration was measured by calcein staining using flow cytometry. The cellular iron marker FTL was measured (*D*) at the mRNA level using RT–PCR and (*E*) at the protein level using Western blotting. Statistical analysis was performed using a *t* test from representative results of three similar experiments. ∗∗∗*p* < 0.001. FTL, ferritin light chain.

**Supplemental Figure S4:**
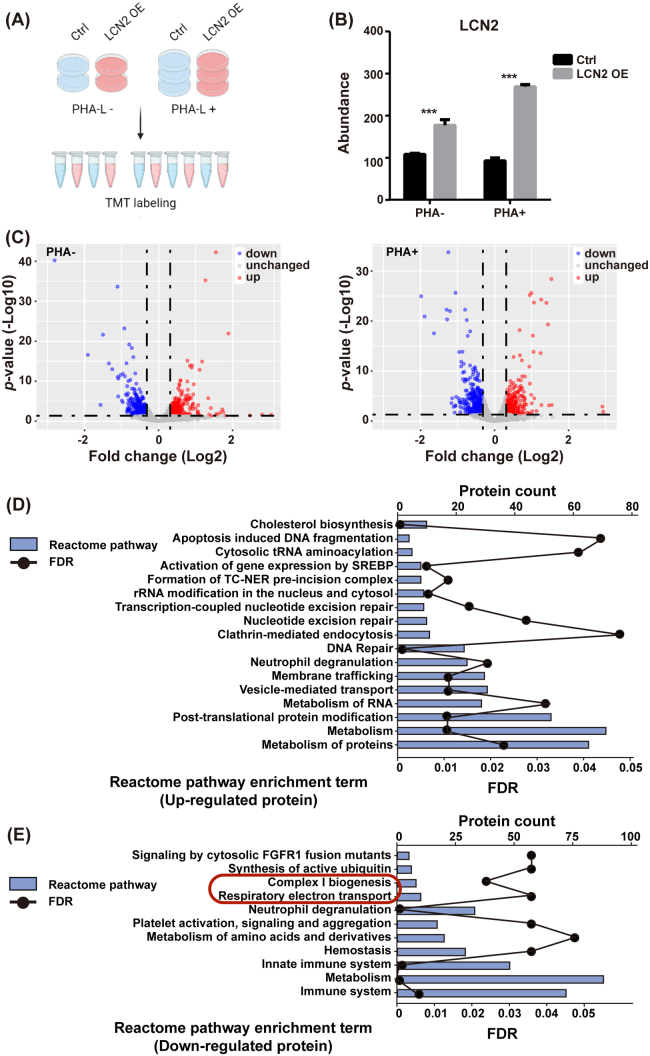


**Supplemental Figure S5:**
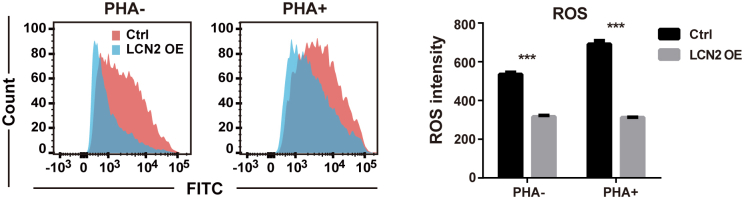


To further explore how LCN2 promotes cell apoptosis, and to comprehensively understand LCN2 functions in T cells, a relative quantitative proteomic study was performed in LCN2-overexpressed Jurkat cells (Supplemental Fig. S4*A*). A total of 6967 proteins were identified (Supplemental Table S7), and LCN2 was overexpressed by 1.64-fold and 2.89-fold without or with PHA treatment, respectively (Supplemental Fig. S4*B*). EdgeR was used to screen DEPs (fold change >1.25 or <0.8; *p* < 0.05) (Supplemental Fig. S4*C*) (29). The DEP list is shown in Supplemental Table S8. To reveal the mechanism underlying LCN2-induced apoptosis, Reactome pathway enrichment was performed on the DEPs (Supplemental Fig. S4, *D* and *E*). Several downregulated proteins were clustered in disrupted respiratory pathways, especially the biogenesis of complex I in the electron transport chain (Supplemental Fig. S4*E*, labeled in *red box*). Our research quantified 7 core subunits and 22 accessory subunits of complex I (Fig. 5*A*), and most quantified accessory subunits were downregulated. In addition, more than 20 assembly factors participate in complex I assembly (47), and of the seven factors quantified in this study, NDUFAF2 and NDUFAF7 were significantly decreased (Fig. 5*A*). Reduced ROS in the LCN2-overexpressed cells reflected the damaged complex I (Supplemental Fig. S5). Meanwhile, several studies have reported the relationship between cellular iron and complex I: iron is important for complex I activity; its deficiency leads to decreased complex I numbers, and exogenous iron supplementation can restore the decreased activity of complex I (48, 49). Importantly, LCN2 is an iron transporter in nature. The cellular iron markers FTL, transferrin receptor, and iron responsive element–binding protein-2 were reduced in our study (Fig. 5*B*), indicating lower cellular iron concentration when LCN2 was overexpressed. Furthermore, cell apoptosis was also confirmed here (Fig. 5*B*). Taken together, we hypothesized that LCN2 induced cell apoptosis by regulating cellular iron concentration.

To verify our hypothesis, we measured cellular iron by calcein staining. Since cellular iron can quench calcein, the low-calcein subgroup of cells is an indicator of cellular iron concentration (50). After LCN2 overexpression, the low-calcein subgroup of cells significantly decreased (Fig. 5*C*), demonstrating that LCN2 reduced cellular iron concentration. The effects of LCN2 on cellular iron were further validated by FTL detection. Our results showed that FTL was downregulated by LCN2 both at the mRNA (Fig. 5*D*) and protein levels (Fig. 5*E*).

**Fig. 6:**
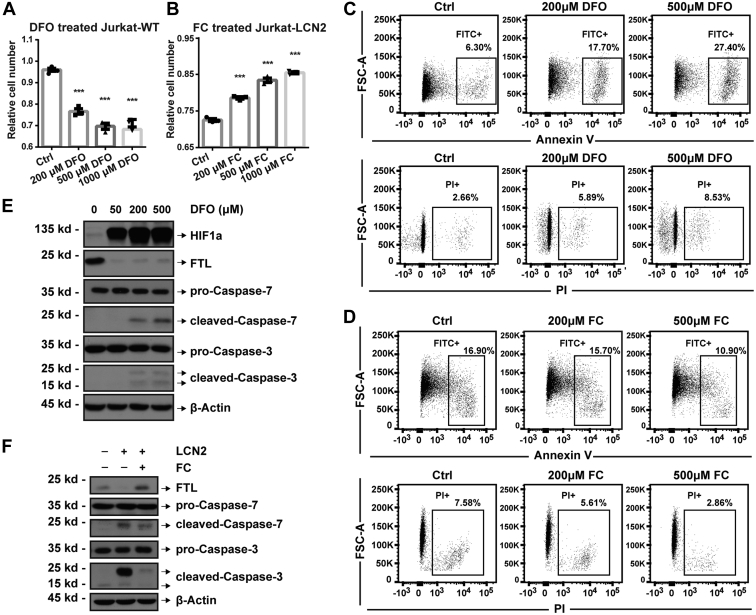
**Cellular iron regulated cell apoptosis in Jurkat cells.** CCK-8 assay was performed in (*A*) iron chelator DFO-treated wildtype Jurkat cells and (*B*) iron supplement FC-treated LCN2-overexpressed Jurkat cells. *C* and *D*, cell apoptosis was measured by annexin V and propidium iodide (PI) signals using flow cytometry after treatments as (*A* and *B*). *E* and *F*, cellular iron (FTL) and cell apoptosis (caspase-7 and caspase-3) were validated at the protein level using Western blotting after treatments as (*A* and *B*). Statistical analysis was performed using a *t* test from representative results of three similar experiments. ∗∗∗*p* < 0.001. CCK-8, Cell Counting Kit-8; DFO, deferoxamine; FC, ferric citrate; FTL, ferritin light chain.

Next, we used the iron chelator DFO to mimic LCN2 overexpression. DFO treatment decreased the number of Jurkat cells (Fig. 6*A*). The iron supplement FC was then used to reverse the effects of LCN2 on Jurkat cells (Fig. 6*B*). DFO treatment enhanced the annexin V/PI signals (Fig. 6*C*). When treated with 500 μM FC, the annexin V and PI signals were reversed by 1.55-fold and 2.65-fold, respectively (Fig. 6*D*). These results were also validated by Western blotting (Fig. 6, *E* and *F*), confirming that LCN2 could induce cell apoptosis by reducing cellular iron.

### LCN2 Promoted Tumor Cell Proliferation *via* its Microenvironment

**Fig. 7:**
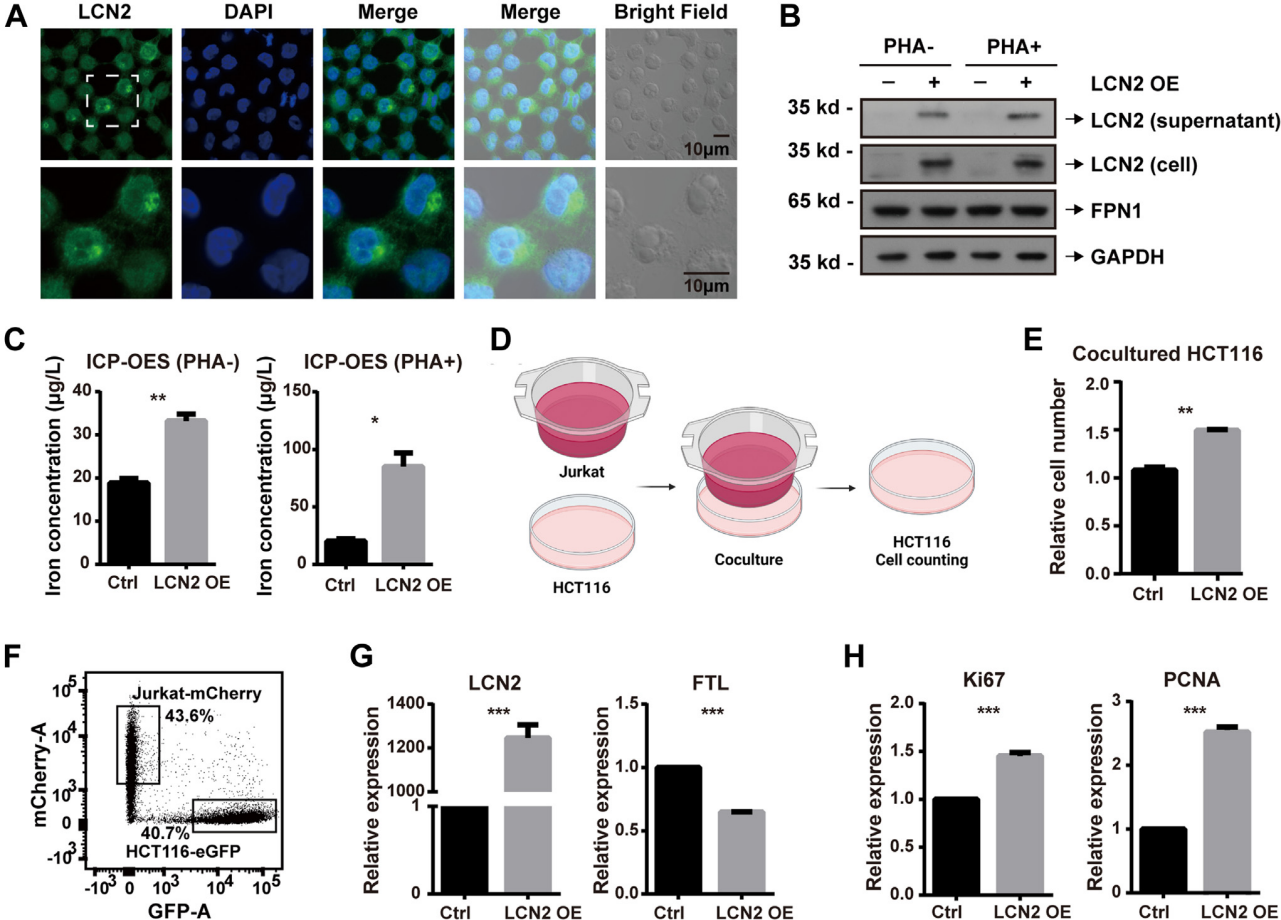
**LCN2 promoted tumor cell proliferation *via* the microenvironment.***A*, LCN2 localization was determined by immunofluorescence (LCN2, *green*; nucleus, *blue*). *B*, LCN2 protein expression in Jurkat cells and in cell supernatant was measured using Western blotting. *C*, iron in the cell supernatant was measured by ICP-OES after culturing for 72 h. *D*, HCT116 cells were cocultured with Jurkat cells in a noncontact coculture system with transwell inserts. After 48 h, (*E*) a CCK-8 assay was performed. *F*, enhanced GFP (eGFP)-labeled HCT116 cells were directly cocultured with mCherry-labeled Jurkat cells, and cells were separated by flow cytometry after 48 h. For separated Jurkat cells, (*G*) overexpressed LCN2 and decreased FTL were validated at the mRNA level using RT–PCR. For separated HCT116 cells, (*H*) the cell proliferation markers Ki67 and PCNA were measured at the mRNA level using RT–PCR. Statistical analysis was performed using a *t* test from representative results of three similar experiments. ∗*p* < 0.05, ∗∗*p* < 0.01, and ∗∗∗*p* < 0.001. CCK-8, Cell Counting Kit-8; FTL, ferritin light chain; ICP-OES, inductively coupled plasma optical emission spectrometry.

Next, we needed to determine where the iron was being transported to cause the reduced iron concentration in the cells. Since secreted LCN2 functions as an iron transporter, we speculated that the iron was being transported outside the cells. Therefore, we verified the secretion and cellular localization of LCN2 in Jurkat cells using immunofluorescence. We found that LCN2 was expressed in the cytoplasm and secreted to the extracellular spaces (Fig. 7*A*). To confirm this result, we performed Western blotting on the Jurkat cells and cell supernatant and detected substantial amounts of LCN2 in both the cells and supernatant (Fig. 7*B*), confirming both the expression and secretion of LCN2. Meanwhile, overexpression of LCN2 did not change the expression of other iron efflux proteins such as FPN1 (Fig. 7*B*). To directly demonstrate the iron efflux of LCN2, ICP-OES was used to measure the iron concentration in the cell supernatant. After 72 h of culturing, overexpressed LCN2 increased the iron concentration in the supernatant by 1.75-fold. When stimulated with PHA, the iron concentration was increased by nearly fourfold (Fig. 7*C*). These results indicated that secreted LCN2 could lead to iron efflux. Therefore, overexpression of LCN2 excreted iron from the cells, leading to a decrease of cellular iron concentration in T cells and an increase of iron concentration in the microenvironment.

**Supplemental Figure S6:**
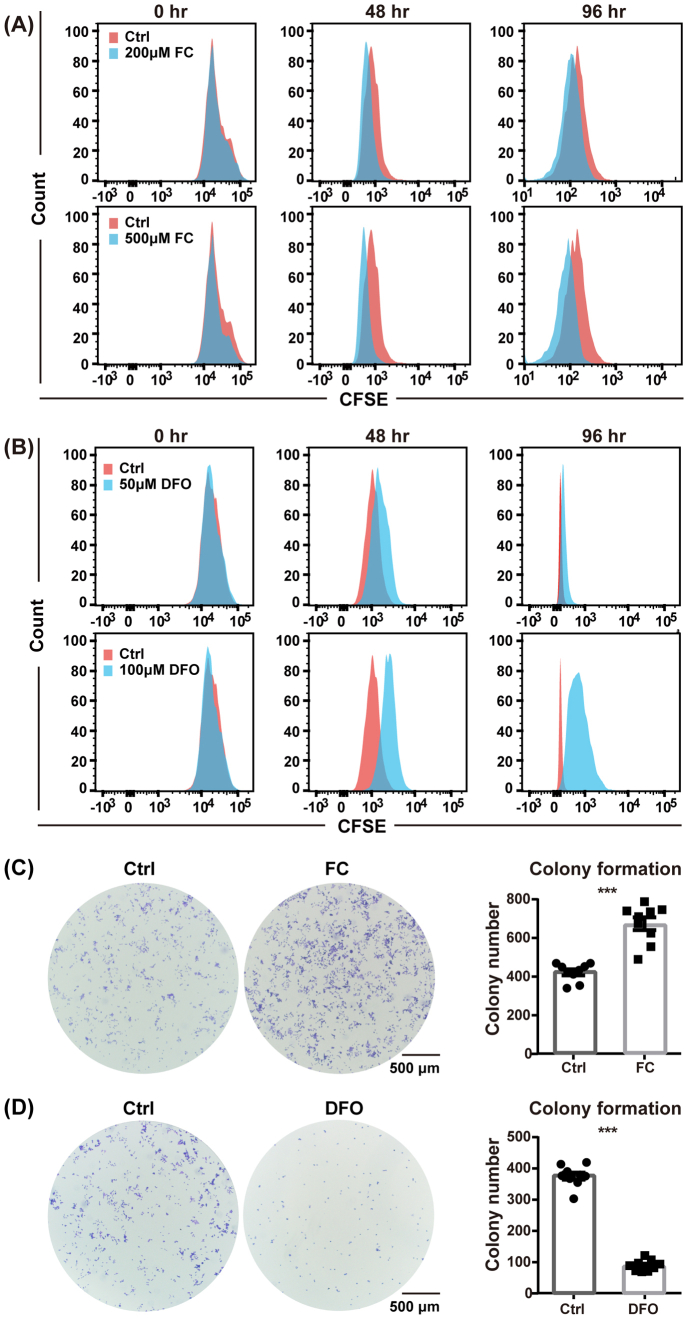


Rapid proliferation of tumor cells is accompanied by higher nutrient requirements in the microenvironment; these nutrients include metal ions such as iron (51). Iron in the microenvironment has been reported to contribute to tumor cell growth (18, 52). In order to verify whether the iron excreted by LCN2 was beneficial to tumor progression, we treated the CRC cell line HCT116 with FC or DFO, and cell proliferation was detected by CFSE staining using flow cytometry. After 48 h of treatment with 200 μM FC, CFSE signals were shifted slightly to the left, and these shifts were more pronounced after 96 h of treatment (Supplemental Fig. S6*A*). Meanwhile, treatment with 100 μM DFO for only 48 h strongly inhibited cell proliferation (Supplemental Fig. S6*B*). The effects of iron on tumor cell proliferation were further validated by colony formation assay (Supplemental Fig. S6, *C* and *D*). These results validated the conclusion that iron in the microenvironment was pivotal for tumor cell proliferation, and that this increased extracellular iron concentration–promoted tumor cell proliferation, which helped tumor development.

Coculture systems were used to mimic the tumor immune microenvironment and to reflect LCN2 functions in tumors. We cocultured HCT116 cells with Jurkat cells using transwell inserts and counted the HCT116 cell number after 48 h (Fig. 7*D*). When cocultured with LCN2-overexpressed Jurkat cells, the number of HCT116 cells increased (Fig. 7*E*), indicating that LCN2 had potential to promote tumor development. Accordingly, we validated this finding using a direct coculture system. We constructed Jurkat cells with mCherry tags and HCT116 cells with enhanced GFP tags to separate the Jurkat and HCT116 cells after coculturing (Fig. 7*F*). For cocultured Jurkat cells, LCN2 and FTL were detected at the mRNA level to ensure the overexpression of LCN2 and efflux of cellular iron (Fig. 7*G*). In the cocultured HCT116 cells, we found that the cell proliferation markers Ki67 and PCNA were increased by LCN2 (Fig. 7*H*), demonstrating that LCN2 promoted HCT116 cell proliferation. These results validated the hypothesis that LCN2 promoted tumor development *via* its microenvironment, which demonstrates the potential use of LCN2 as an immunotherapy target.

### LCN2 Affected Cholesterol Metabolism in Lymphocytes

**Supplemental Figure S7:**
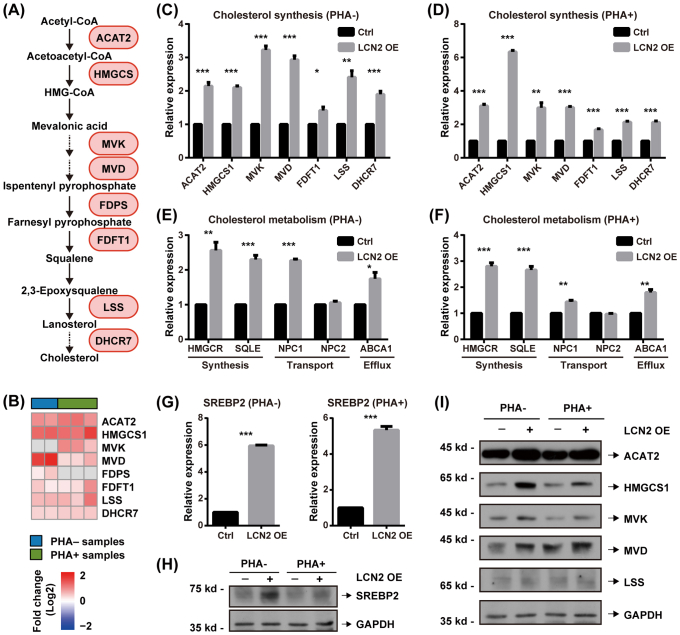


In addition to iron transport, and its effects on lymphocyte apoptosis and tumor cell proliferation, the relative quantitative proteomic study of the LCN2-overexpressed cell line showed other functions of LCN2 in CRC. The pathway enrichment analysis results showed enrichment in cholesterol biosynthesis and activation of gene expression by SREBP (Supplemental Fig. S4*D*). Specifically, eight significantly enhanced proteins (ACAT2, HMGCS, MVK, MVD, FDPS, FDFT1, LSS, and DHCR7) participated in cholesterol biosynthesis (Supplemental Fig. S7, *A* and *B*), and their differential expression was validated at the mRNA level (Supplemental Fig. S7, *C* and *D*). Meanwhile, we discovered that the expression of several other genes related to cholesterol metabolism was also substantially altered, such as HMGCR and SQLE in cholesterol synthesis, NPC1 and NPC2 in cholesterol trafficking, and ABCA1 in cholesterol efflux (Supplemental Fig. S7, *E* and *F*). The cholesterol metabolism regulator SREBP2 increased because of LCN2 overexpression at both the mRNA and protein levels (Supplemental Fig. S7, *G* and *H*). In addition, the eight significantly enhanced proteins in Supplemental Fig. S7*A* were validated at the protein level (Supplemental Fig. S7*I*). We were curious whether these changes in cholesterol metabolism had similar effects on tumor cells as cellular iron. Therefore, we measured the total cholesterol in both the cells and cell supernatant, which revealed that the increased cholesterol was distributed in the cell supernatant instead of accumulating in the cells (Supplemental Fig. S8, *A* and *B*). Cholesterol promotes tumor metastasis by affecting cell stemness or cell membrane fluidity (53, 54), which our research validated. Since cholesterol exists primarily as LDL-C and high-density lipoprotein-cholesterol, and LDL-C accounted for >75% of the total, we tested the effects of cholesterol on tumor cells using LDL-C and total cholesterol treatments. A cell invasion assay was performed using transwell inserts with Matrigel, revealing that only LDL-C promoted cell invasion (Supplemental Fig. S8, *C* and *D*). Collectively, these results indicated that LCN2 promoted tumor metastasis.

## Discussion

Although there is a consensus that T cells play important roles in antitumor immune responses, such as the CIC, few studies have focused on a comprehensive proteomic analysis of tumor-infiltrating T cells. Since proteins are direct executors of functions, we believe that proteomic studies not only have a stronger correlation with tumor progression than genomic studies but also have a greater clinical value for diagnosis and treatment. However, studies describing the detailed characteristics of tumor-infiltrating T cells have primarily been performed at the genome level (55, 56, 57). Moreover, most proteomic studies are based on whole-tissue samples instead of sorting cell subgroups (58, 59, 60), which will miss the unique protein expression pattern of a specific cell subgroup. Therefore, proteomic studies of sorted tumor-infiltrating T cells have greater value in revealing the functions and describing the characteristics of T cells in the tumor immune microenvironment. Accordingly, the screening of DEPs may provide new targets for antitumor therapies, which has great prospects for clinical applications. Moreover, contrary to most omics research that ends at the bioinformatic analysis, our study further explored the functions and mechanism of LCN2 in cell models, which mutually verified the two parts of our study, thereby improving the credibility of our research results.

To our knowledge, this is the first relative quantitative proteomic study of tumor-infiltrating T cells, particularly in CRC. Therefore, this study provides advanced quantitative information at the protein level in this field. Our proteomic study of clinical samples identified 7310 proteins—far more than previous studies—providing a superior amount of data as the basis of this study relative to similar studies (61, 62). To avoid the noise of irrelevant factors, DNTs were paired with corresponding tumor tissues for comparisons, and comprehensive quality evaluations of the dataset were performed prior to subsequent analysis. Among the 258 screened DEPs, eight known targets were listed in the top 20 DEPs with the largest fold changes, including B2M (23, 24), CD36 (29), CEACAM6 (31), and five members from the S100 family (33, 34, 35, 36, 37), which confirmed the reliability of this study. Bioinformatics analyses, such as GO enrichment, pathway enrichment, and PPI network analysis, were performed, which revealed characteristics of T cells in CRC at the protein level for the first time. Specifically, T cells in CRC had more active ribosomes, increased production of immunosuppressive factors, and disrupted cell homeostasis and ECM organization. These results provide a theoretical basis for further studies in related fields. In addition, the other key DEPs we screened also exhibit great prospects for further exploration.

Most LCN2 research has focused on tumor cells (18, 63); although a few studies have reported its expression and functions in other immune cells such as macrophages (52, 64), its involvement in T cells remains unclear. In this study, LCN2 was screened for the first time as a key DEP in tumor-infiltrating T cells, particularly in CD4^+^ T cells. In addition, we clarified the molecular mechanism of LCN2 functions in tumor-infiltrating T cells. Tumor cells overexpressing LCN2 have been reported to have iron-capturing functions in leptomeningeal metastasis (18) or to develop resistance to ferroptosis inducers in CRC (63). However, our study demonstrated that LCN2 functioned as an iron-disturbing factor to not only induce T-cell apoptosis but also to remove iron from the T cells for use by the tumor cells, thereby enhancing tumor cell proliferation and promoting tumor progression. Moreover, the relative quantitative proteomic study of LCN2-overexpressed Jurkat cells expanded the known roles of LCN2. In addition to iron regulation, bacteriostasis (11), and epithelial–mesenchymal transition regulation (20), we found that LCN2 damaged the antitumor immunity and promoted cholesterol metabolism in T cells, which may be beneficial to tumor metastasis. However, these two functions of LCN2 require further exploration.

Much work remains to validate the feasibility of LCN2 to serve as a specific immunotherapy target for CRC. Conditional knockout mouse models should be used to confirm whether LCN2 silencing has the potential to resist CRC development. Furthermore, there are no drugs currently available targeting LCN2 or inhibitors to suppress the functions of LCN2. Therefore, to test the potential of LCN2 as a drug target, these drugs will need to be developed. Current antibodies against LCN2 provide only partial inhibition, since LCN2 is a secreted protein than can be produced and secreted from cells continuously. As a member of the lipocalin family, LCN2 has a pocket at the core of its structure, which is pivotal for its functions (10). Therefore, this pocket should be considered when designing its inhibitors, which will be a focus of our future research projects.

## Conclusions

In summary, we collected tumor tissues and paired DNTs from CRC patients, sorted T cells from the tissues, and performed a relative quantitative proteomic study by TMT labeling. Bioinformatic analysis was used to describe the protein expression pattern of T cells, revealing characteristics of tumor-infiltrating T cells and screening key DEPs. Upregulated LCN2 was screened from T cells in CRC, and further exploration was accomplished using a Jurkat cell model. We demonstrated that the iron transporter LCN2 induced lymphocyte apoptosis by reducing cellular iron concentration. Iron efflux increased iron concentrations in the tumor microenvironment, which aided tumor cell proliferation and promoted tumor development. Our research validated the potential use of LCN2 as an immunotherapy target.

## Ethics Statement

The studies involving human participants were reviewed and approved by the Ethics Commission of Beijing Chao-Yang Hospital. The patients/participants provided their written informed consent to participate in this study.

## Data Availability

The MS proteomics data have been deposited to the ProteomeXchange Consortium (http://proteomecentral.proteomexchange.org) *via* the iProX partner repository with the dataset identifier PXD041366.

Figures were created with biorender.com.

## Supplemental data

This article contains supplemental data.

## Conflict of interest

The authors declare no competing interests.

## Figures and Tables

**Supplemental Figure S8:**