# Supplementary material for: Quantitative Proteomics of Tissue-Infiltrating T Cells From CRC Patients Identified Lipocalin-2 Induces T-Cell Apoptosis and Promotes Tumor Cell Proliferation by Iron Efflux
Source: Mol Cell Proteomics. 2023 Dec 10;23(1):100691. doi: 10.1016/j.mcpro.2023.100691 (PMC10792491; doi:10.1016/j.mcpro.2023.100691)
Supplement: Supplemental Data [file mmc1.docx]

**Quantitative proteomics of tissue-infiltrating T cells from CRC patients reveals that Lipocalin-2 induces T cell apoptosis and promotes tumor cell proliferation by iron efflux**

Rui Che^1^, Qingsong Wang^1^, Minzhe Li^2^, Jian Shen^2^*, Jianguo Ji^1^*

1. Material List

**F_IG_. S1. PPI network analysis of the DEPs in T cells from CRC patient tumors.**

**F_IG_. S2. Validation of LCN2-overexpressed cell line and PHA stimulation.**

**F_IG_. S3. LCN2 reduced cell number with no effect on cell proliferation in various leukemia cell lines.**

**F_IG_. S4. proteomic study of LCN2-overexpressed Jurkat cells.**

**F_IG_. S5. LCN2 disturbed ROS production in Jurkat cells.**

**F_IG_. S6. Iron in the microenvironment regulated tumor cell proliferation.**

**F_IG_. S7. LCN2 activated cholesterol metabolism and promoted cholesterol metabolism in Jurkat cells.**

**F_IG_. S8. LDL-C promoted tumor cell invasion.**

**Table S1.** TMT labeling strategy of T cell samples from CRC patients.

**Table S2.** TMT labeling strategy of LCN2-overexpressed Jurkat cells.

**Table S3.** Clinical information of CRC patient subjects.

**Table S4.** Primers for RT-PCR.

**Table S5.** Quantitative proteomic data of T cell samples from CRC patients.

**Table S6.** DEPs in quantitative proteomic data of T cell samples from CRC patients.

**Table S7.** Quantitative proteomic data of LCN2-overexpressed Jurkat cells.

**Table S8.** DEPs in quantitative proteomic data of LCN2-overexpressed Jurkat cells.

1. Supplemental Figure Legends


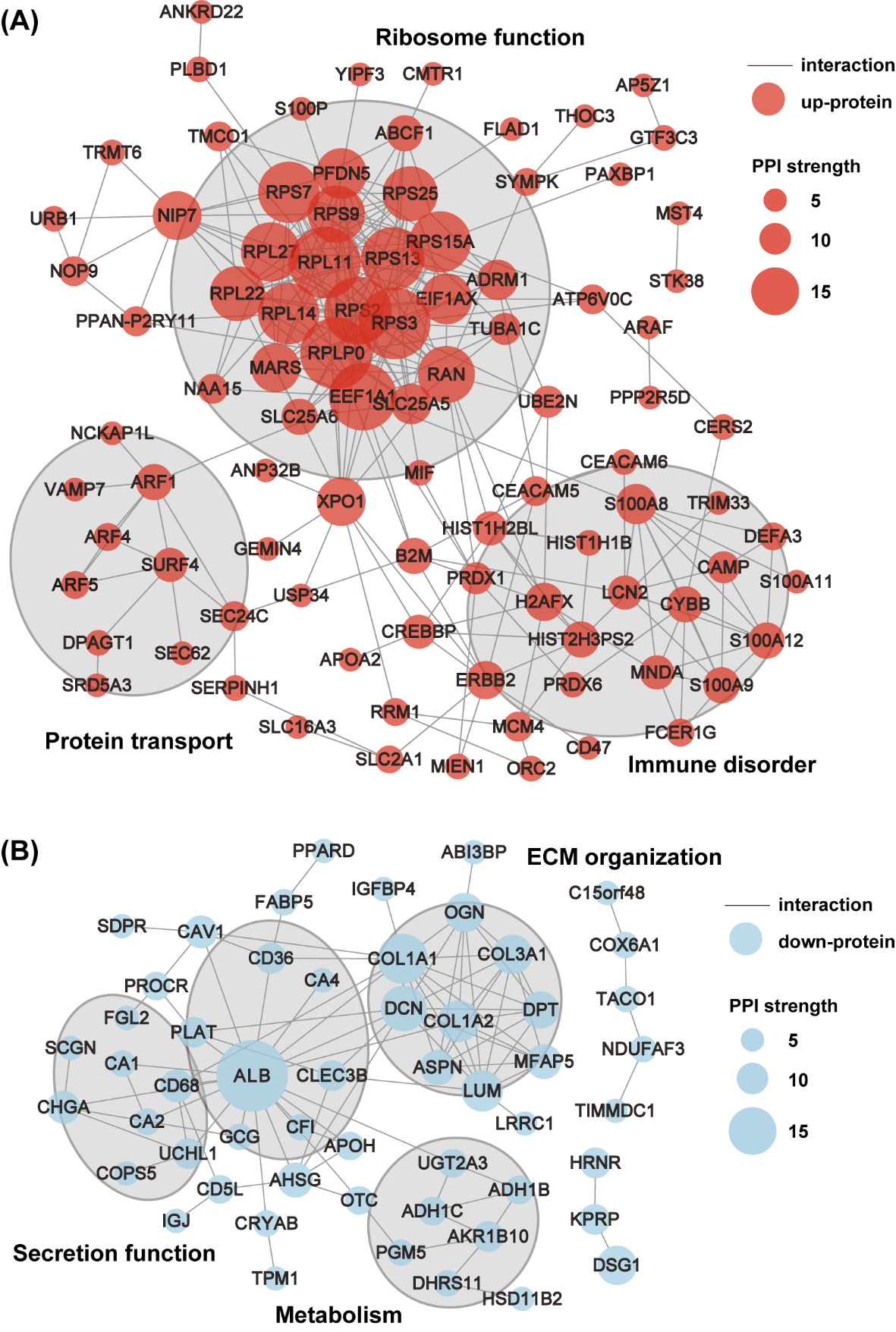


**F_IG_. S1. PPI network analysis of the DEPs in T cells from CRC patient tumors.** PPI network analysis of significantly **(*A*)** up-regulated proteins (red) and **(*B*)** down-regulated proteins (blue). Grey lines indicate interactions. Abbreviations: PPI, protein-protein interaction; DEPs, differentially expressed proteins; CRC, colorectal cancer.


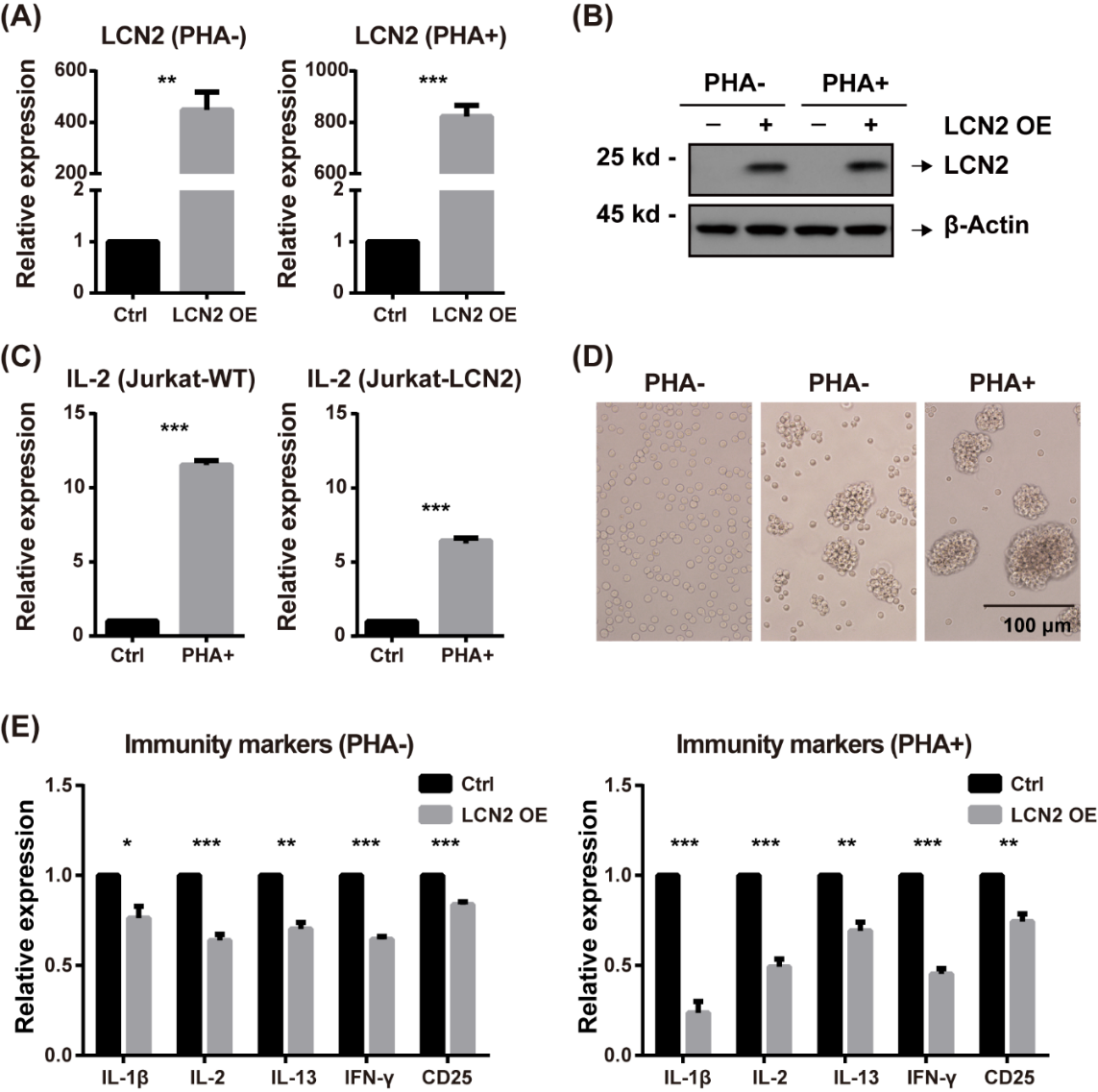


**F_IG_. S2. Validation of LCN2-overexpressed cell line and PHA stimulation.** LCN2 was stably overexpressed in Jurkat cells, and the overexpression of LCN2 was validated **(*A*)** at the mRNA level using RT-PCR and **(*B*)** at the protein level using western blotting. **(*C*)** IL-2 measurement using RT-PCR and **(*D*)** morphological observation were performed to confirm the stimulation and activation of Jurkat cells after 2 μg/mL PHA treatment for 48 hr. **(*E*)** Immune cytokines and T cell activation marker was measured in Jurkat cells at the mRNA level using RT-PCR. Statistical analysis was performed using a *t*-test from representative results of three similar experiments. **p*<0.05, ***p*<0.01, ****p*<0.001. Abbreviation: PHA, Phytohemagglutinin.


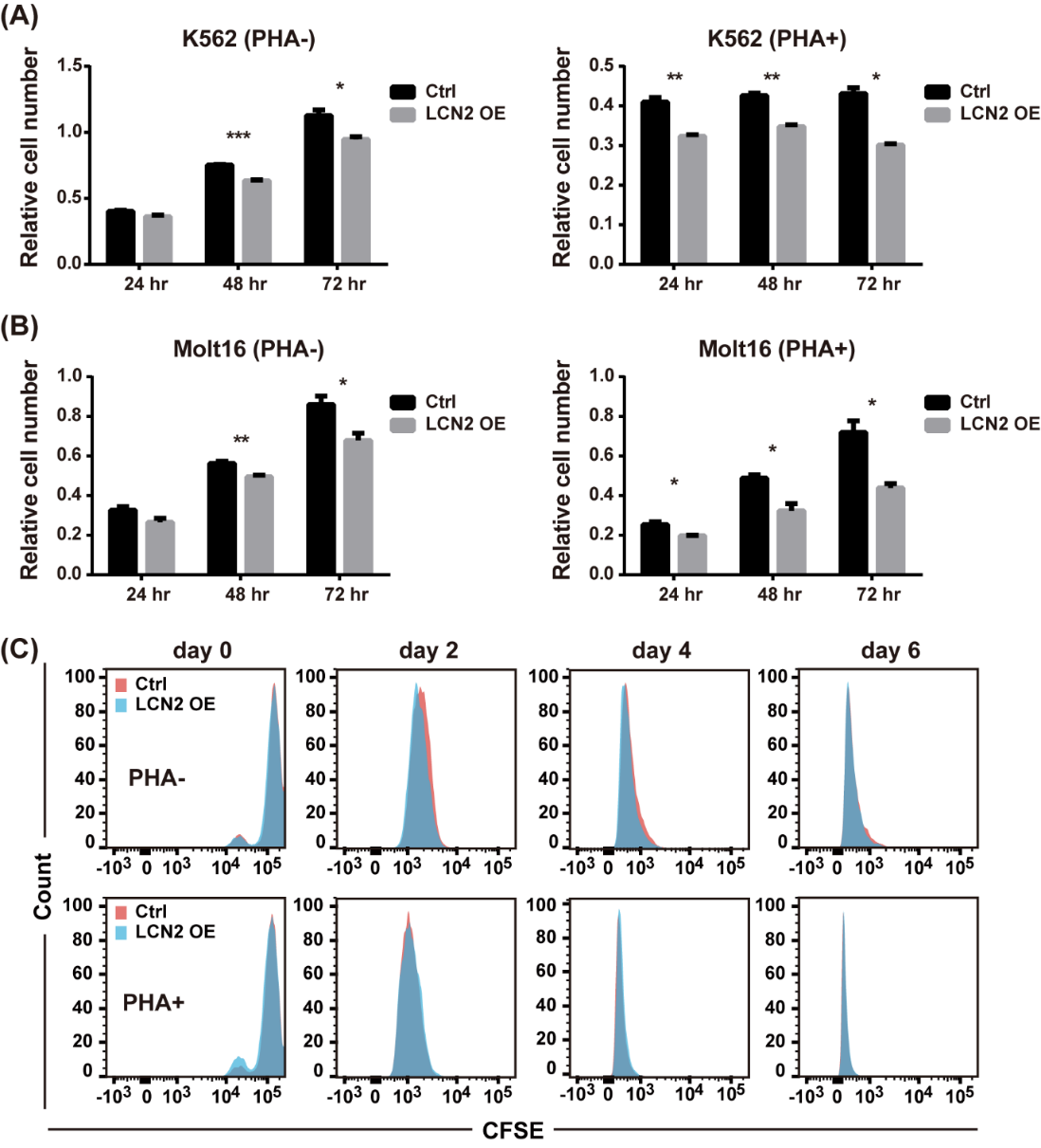


**F_IG_. S3. LCN2 reduced cell number with no effect on cell proliferation in various leukemia cell lines.** To verify the results seen in Jurkat cells in response to LCN2 overexpression, a CCK-8 assay was similarly performed in **(*A*)** K562 cells and **(*B*)** Molt16 cells. **(*C*)** Cell proliferation was measured by CFSE staining using flow cytometry. Statistical analysis was performed using a *t*-test from representative results of three similar experiments. **p* < 0.05, ***p*<0.01, ****p*<0.001. Abbreviation: CCK-8, Cell Counting Kit-8.


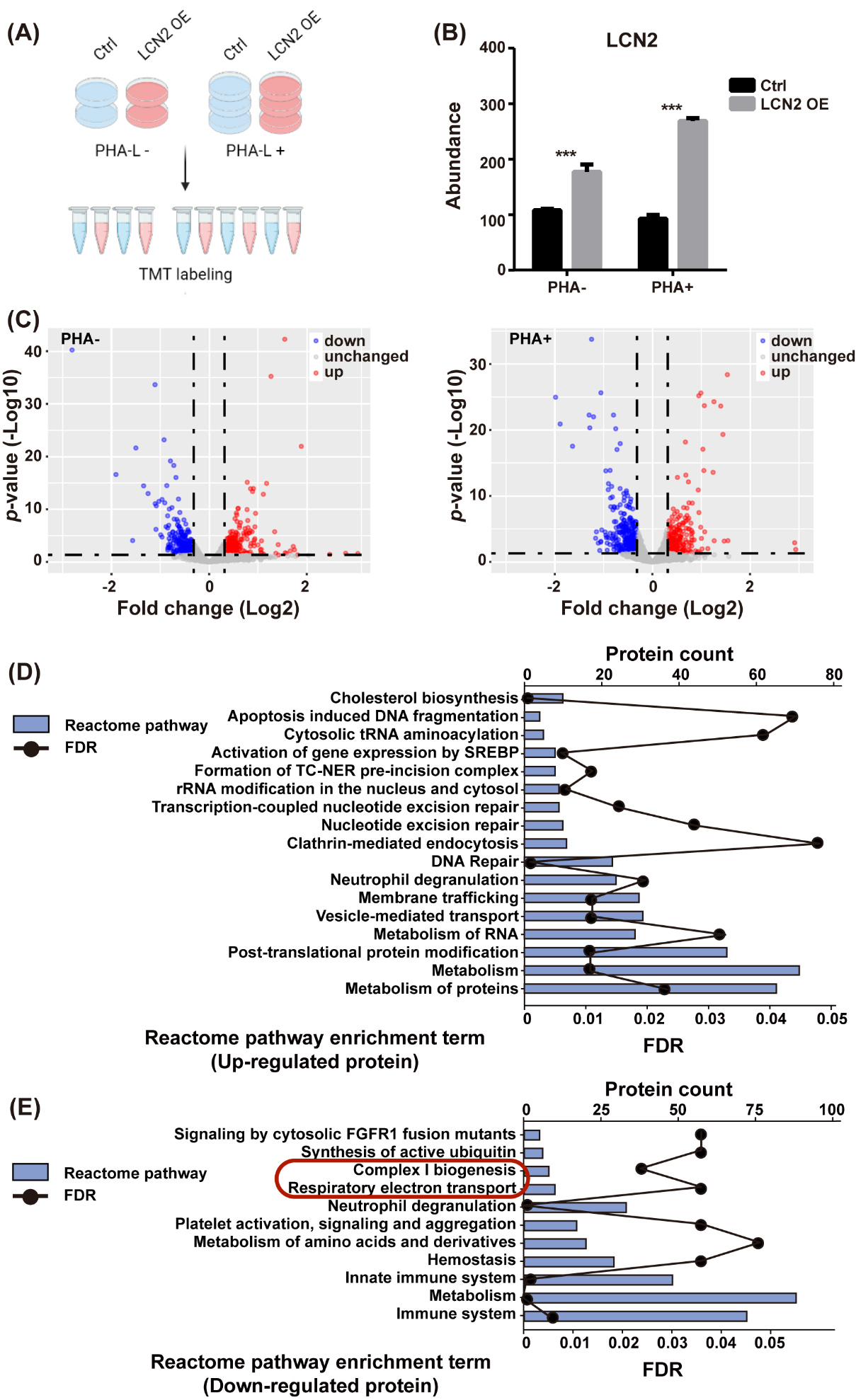


**F_IG_. S4. proteomic study of LCN2-overexpressed Jurkat cells. (*A*)** A relative quantitative proteomic study of LCN2-overexpressed Jurkat cells was accomplished using TMT labeling. Figures were created with biorender.com. **(*B*)** Overexpression of LCN2 was confirmed in this proteomic study. **(*C*)** DEPs in LCN2-overexpressed Jurkat cells without or with PHA stimulation were visualized using volcano plots, with significantly up-regulated proteins (fold change >1.25, *p*<0.05) in red and down-regulated proteins (fold change <0.80, *p*<0.05) in blue. Reactome pathway enrichment analysis of significantly **(*D*)** up-regulated and **(*E*)** down-regulated proteins. For the enrichment results with more than 20 terms, only the top 20 terms with the strongest enrichment intensity are shown here. Statistical analysis was performed using a *t*-test of LCN2-overexpressed cells (LCN2 OE) to wild-type Jurkat cells (Ctrl). ****p*<0.001. Abbreviations: DEPs, differentially expressed proteins; PHA, Phytohemagglutinin.


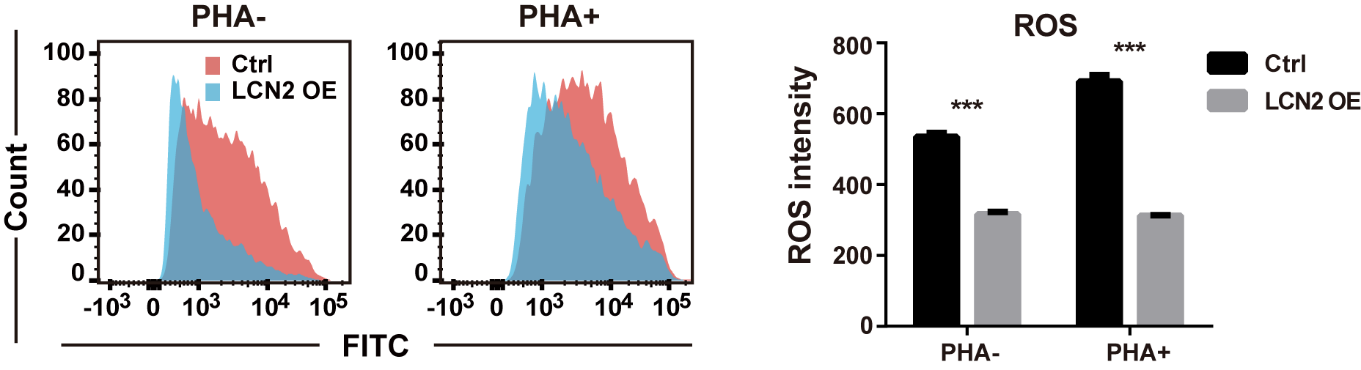


**F_IG_. S5. LCN2 disturbed ROS production in Jurkat cells.** ROS production was measured by DCFH-DA probes using flow cytometry and ELISA. Statistical analysis was performed using a *t*-test from representative results of three similar experiments. ****p*<0.001. Abbreviation: ROS, reactive oxygen species.


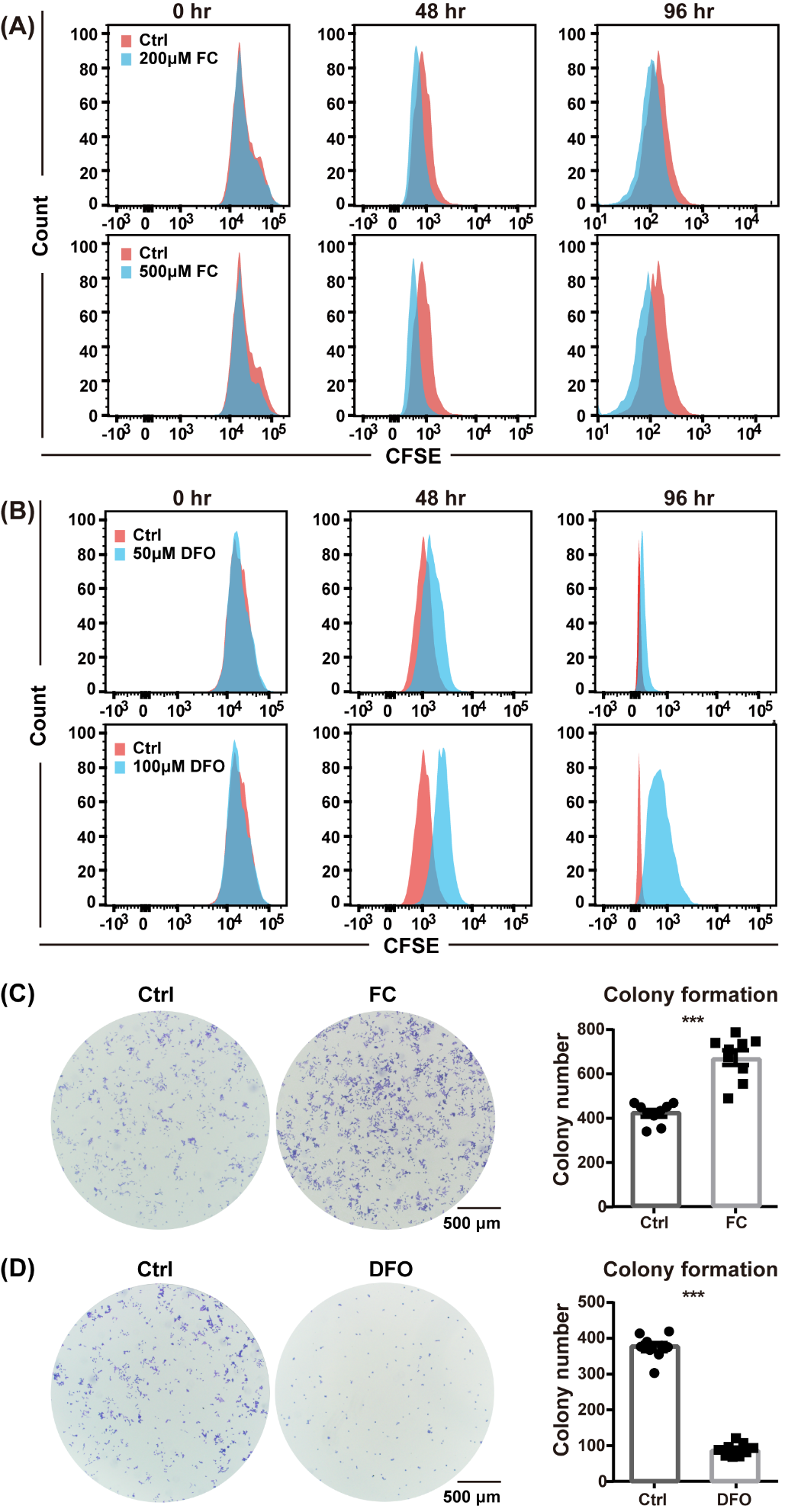


**F_IG_. S6. Iron in the microenvironment regulated tumor cell proliferation.** Cell proliferation was measured by CFSE staining using flow cytometry on CRC cell line HCT116 treated with different concentrations of **(*A*)** FC for 48 hr or **(*B*)** DFO for 48 hr. For the colony formation assay, HCT116 cells were treated with **(*C*)** 500 μM FC or **(*D*)** 200 μM DFO and colony numbers were counted after 7 day. Statistical analysis was performed using a *t*-test from representative results of three similar experiments. ****p*<0.001. Abbreviations: CRC, colorectal cancer; DFO, Deferoxamine; FC, Ferric citrate.


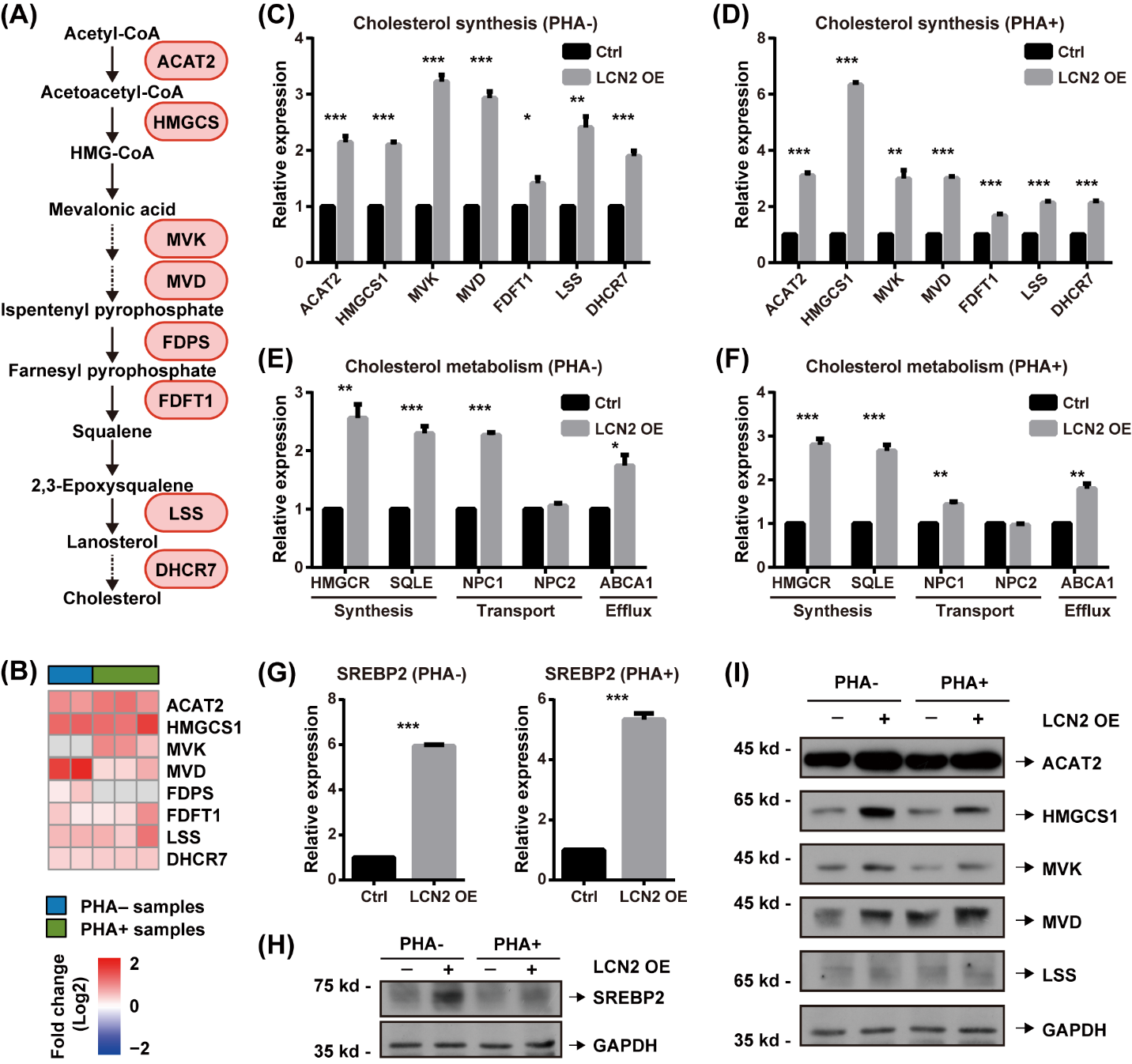


**F_IG_. S7. LCN2 activated cholesterol metabolism and promoted cholesterol metabolism in Jurkat cells. (*A*)** Several proteins that were up-regulated in LCN2-overexpressed Jurkat cells were related to cholesterol biogenesis. **(*B*)** Expression of those cholesterol biogenesis-related proteins are visualized by heatmap. Proteins related to cholesterol synthesis revealed by proteome data were validated again **(*C-D*)** at the mRNA level using RT-PCR. Other genes related to cholesterol metabolism were detected **(*E-F*)** at the mRNA level using RT-PCR. The cholesterol metabolism regulator SREBP2 was measured **(*G*)** at the mRNA level using RT-PCR and **(*H*)** at the protein level using western blotting. **(*I*)** Proteins in **(*A*)** were validated at the protein level using western blotting. Statistical analysis was performed using a *t*-test from representative results of three similar experiments. **p*<0.05, ***p*<0.01, ****p*<0.001.


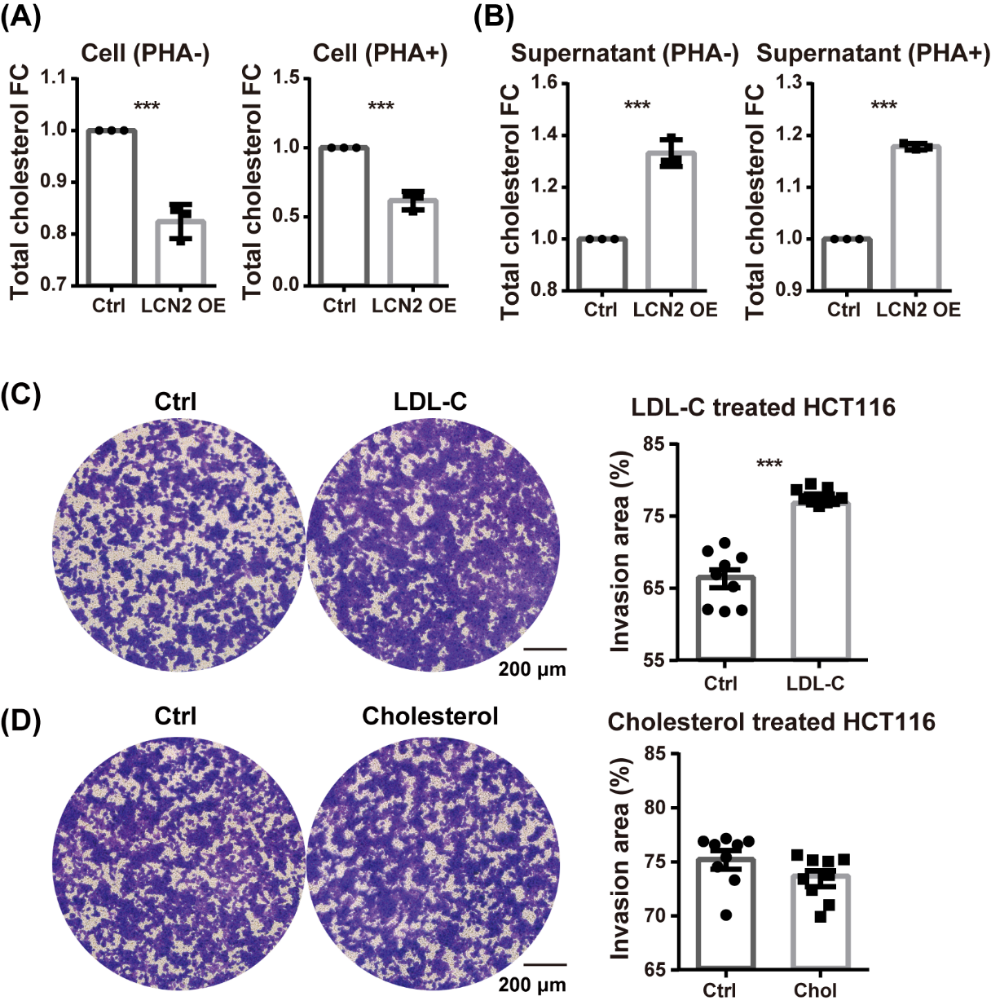


**F_IG_. S8. LDL-C promoted tumor cell invasion.** Total cholesterol **(*A*)** in Jurkat cells and **(*B*)** in the cell supernatant were measured using ELISA. A cell invasion assay was performed with crystal violet staining after treatments with **(*C*)** LDL-C and **(*D*)** total cholesterol. Statistical analysis was performed using a *t*-test from representative results of three similar experiments. ****p*<0.001. Abbreviations: LDL-C, Low-density lipoprotein-cholesterol; chol, cholesterol.

1. Supplemental Tables

**Table S1.** TMT labeling strategy of T cell samples from CRC patients.

**Table S2.** TMT labeling strategy of LCN2-overexpressed Jurkat cells.

**Table S3.** Clinical information of CRC patient subjects.

**Table S4.** Primers for RT-PCR.

**Table S5.** Quantitative proteomic data of T cell samples from CRC patients.

**Table S6.** DEPs in quantitative proteomic data of T cell samples from CRC patients.

**Table S7.** Quantitative proteomic data of LCN2-overexpressed Jurkat cells.

**Table S8.** DEPs in quantitative proteomic data of LCN2-overexpressed Jurkat cells.
